# Hippocampal representation during collective spatial behaviour in bats

**DOI:** 10.1038/s41586-023-06478-7

**Published:** 2023-08-30

**Authors:** Angelo Forli, Michael M. Yartsev

**Affiliations:** 1 Department of Bioengineering, UC Berkeley, Berkeley, CA USA; 2 Helen Wills Neuroscience Institute, UC Berkeley, Berkeley, CA USA

**Keywords:** Hippocampus, Social behaviour

## Abstract

Social animals live and move through spaces shaped by the presence, motion and sensory cues of multiple other individuals^1–6^. Neural activity in the hippocampus is known to reflect spatial behaviour^7–9^ yet its study is lacking in such dynamic group settings, which are ubiquitous in natural environments. Here we studied hippocampal activity in groups of bats engaged in collective spatial behaviour. We find that, under spontaneous conditions, a robust spatial structure emerges at the group level whereby behaviour is anchored to specific locations, movement patterns and individual social preferences. Using wireless electrophysiological recordings from both stationary and flying bats, we find that many hippocampal neurons are tuned to key features of group dynamics. These include the presence or absence of a conspecific, but not typically of an object, at landing sites, shared spatial locations, individual identities and sensory signals that are broadcasted in the group setting. Finally, using wireless calcium imaging, we find that social responses are anatomically distributed and robustly represented at the population level. Combined, our findings reveal that hippocampal activity contains a rich representation of naturally emerging spatial behaviours in animal groups that could in turn support the complex feat of collective behaviour.

## Abstract (web summary)

In bats engaged in spontaneous collective spatial behaviour, a robust spatial structure emerges at the group level whereby behaviour is anchored to specific locations, movement patterns and individual social preferences, and many hippocampal neurons are tuned to key features of group dynamics.

## Main

Many animals, including humans, naturally live, forage and negotiate spaces occupied by other group members^1–6^. In such settings, a fixed spatial layout can embed a highly dynamic sociospatial environment, in which positions and movement patterns of individuals can vary widely from one moment to the next. Navigating such complex environments necessitates keeping track of one’s own position as well as the positions of many other group members, their shared spatial locations (such as roosting spots and food sources) and the sensory signals that are broadcasted by conspecifics. The mammalian hippocampus is believed to represent positional information^7,8^, yet its relationship to spatial behaviour in social settings has largely been studied in single animals or pairs^10,11^ and typically under constrained behavioural paradigms^12–18^. Such settings preclude access to core ethological features of collective behaviours that often occur spontaneously in groups, are self-organized and depend on the identity-specific preferences of individuals^1,5,6^. To bridge this gap, we took advantage of the natural behaviour of Egyptian fruit bats, an extraordinary social mammal that lives, moves and forages in groups^19^. Members of this species spend nearly the entirety of their lives negotiating spaces alongside many other conspecifics^2,3,20^, where they develop clear social^21,22^ and spatial preferences^3,23^ that can collectively guide group behaviour.

## Self-organized collective behaviour in bats

**Fig. 1:**
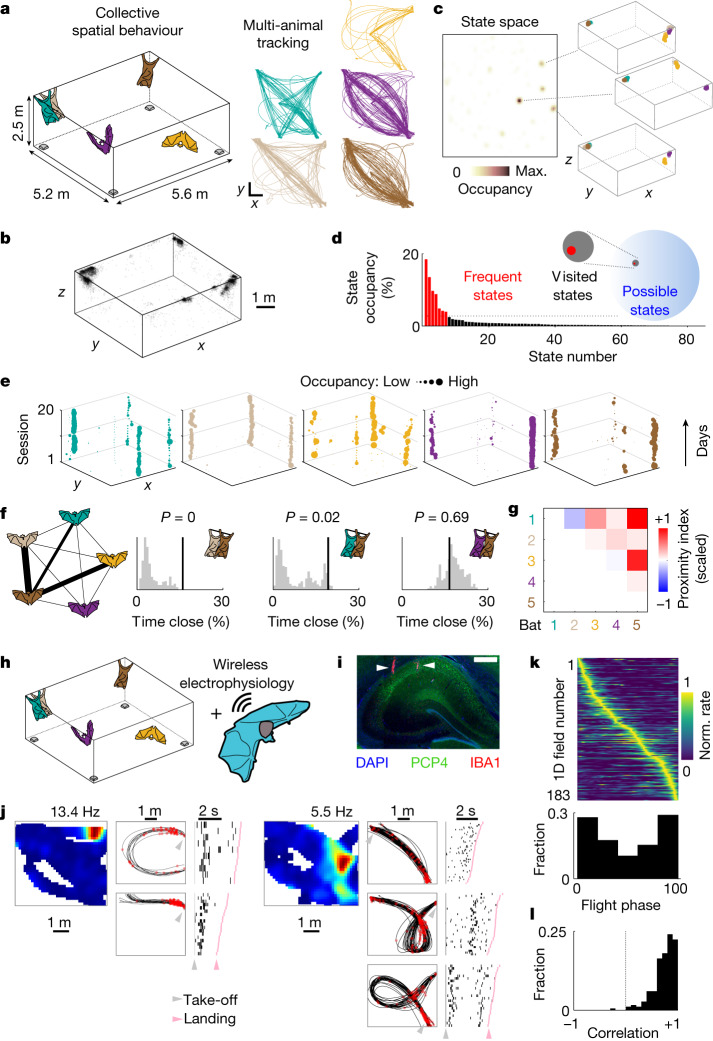
Collective spatial behaviour and hippocampal electrophysiology in groups of bats. **a**, Left, schematic of the experiment: groups of bats were tracked by a RTLS (sample devices shown in grey). Right, the tracked positions of five bats during a representative session (different colours, top view). In this case, a bowl of food (banana) was placed in the centre of the room. Scale bars, 1 m. **b**, The resting positions from all of the bats and sessions (random subsample of 7,685 points; reward locations are excluded). **c**, Colour-coded occupancy in the state space for one representative session: each state corresponds to a configuration of five bats. The three most common configurations are shown on the right (different bats are indicated by different colours). Max., maximum. **d**, The state occupancy distribution for the same session shown in **c**. Frequent states (Methods) are indicated in red. Inset, scaled representation of the number of possible, visited and frequent states for that session. Note that, as a group, an extremely limited fraction of all of the possible states is occupied. **e**, Preferred resting locations (*xy* projection) of each bat (different colours) across consecutive sessions (vertical axis) involving the same group. The marker size was scaled to occupancy. **f**, Left, schematic of the social structure from one representative session. The edge thickness is proportional to the significance of social proximity (Methods; thick edges, *P* < 0.001, *P* < 0.01 and *P* < 0.05; thin edges, not significant). Right, empirical values (black lines), shuffled distributions (grey histograms) and associated *P* values (top) for the percentage of time that specific bat pairs spent in close contact. The pairs are from the graph on the left. **g**, Matrix of average proximity indexes (Methods; scaled to the maximal observed value) for the same group and sessions shown in **e**. **h**, Schematic of the experimental paradigm for wireless electrophysiology recordings during collective behaviour. **i**, A coronal section of the dorsal hippocampus in one recorded bat, stained for 4′,6-diamidino-2-phenylindole (DAPI), PCP4 and IBA1 (Methods). The white arrows denote tetrode tracks. Scale bar, 500 µm. Tetrode localization in the hippocampus was histologically confirmed for each recorded bat (*n* = 5 bats). **j**, Spatial firing of two example cells recorded in the group context. 2D firing field (top view, peak firing rate indicated) is shown on the left. Firing on repeated flight paths is shown both in space (middle; trajectories are shown in black and spikes are shown in red) and time (right; raster plots, sorted by flight duration relative to take-off and landing). **k**, Top, the trial-averaged activity from all significant firing fields recorded on flight paths across bats, rescaled from take-off to landing and sorted by location of peak activity. Bottom, the distribution of the fields’ peak location as a function of flight phase (0 is take-off, 100 is landing) for the same 1D fields shown on top (*n* = 183 1D fields from 95 cells, 3 bats). Norm., normalized. **l**, Correlation values between firing fields calculated on random halves of the trials, for the same fields as in **k**. Source Data

**Extended Data Fig. 1:**
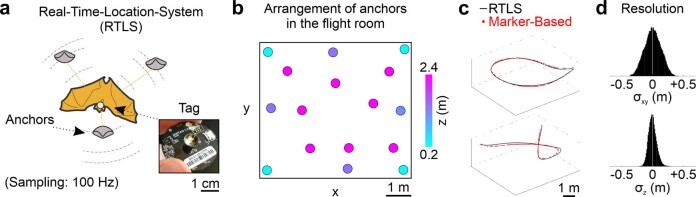
Real-Time-Location-System for tracking multiple animals. **a**, Schematic depiction of the Real-Time-Location-System. A set of static anchors (grey) communicate with a mobile tag (inset) for localization. The tag is mounted on a collar, carried by a bat. **b**, Arrangement of the anchors in the experimental room: eight anchors are mounted on the ceiling (purple), four on the walls (blue) and four on the ground (cyan). **c**, Two example flights executed by a bat simultaneously tracked with the RTLS system and a marker-based system with millimetre-resolution (Methods). **d**, Distribution of tracking errors in the xy (parallel to the floor) and z (vertical) dimensions. Tracking errors are calculated as the difference between the RTLS position and the marker-based position (n = three bats tracked by both systems across eight sessions), after rigid registration of the coordinate systems.

**Extended Data Fig. 2:**
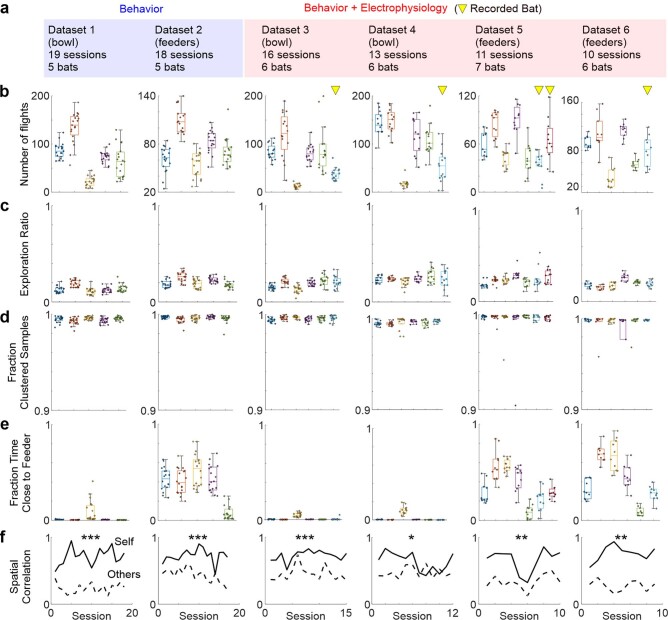
Group foraging datasets and associated behavioural quantifications. **a**, Description of the main datasets included in this study. From top to bottom, each row indicates name of the dataset, food source (Methods), total number of recorded sessions, total number of bats simultaneously involved in the daily sessions. **b**, Number of flights executed by individual bats (different colours) across the corresponding dataset (a). For this panel, as well as for **c-e**, single session values are reported (markers), together with summary statistics for each bat (box: median, lower, and upper quartiles; lines: minimum and maximum values that are not outliers, ‘n’ corresponds to the number of sessions for each bat in the examined dataset). **c**, Exploration ratio (fraction of visited locations, Methods) of individual bats across the sessions from the corresponding dataset (a). **d**, Fraction of samples during rest that were part of a clustered location (Methods) for individual bats across the sessions from the corresponding dataset (a). **e**, Fraction of time spent close to a feeding location, for individual bats across the sessions from the corresponding dataset (a). **f**, Average spatial correlation between occupancy maps of individual bats on consecutive sessions (session j and session j+1; solid line) and average spatial correlation between occupancy maps from different bats on the same session (dashed line). Data are shown for all the sessions associated with a given dataset (a). Stars above the plot indicate the significance level of the difference between self-correlations and others-correlations (*p* = 2 x10^−4^, 4 x10^−4^, 6 x10^−5^, 0.016, 2 x 10^−3^, 4 x10^−3^, Wilcoxon signed rank test, ***: *p* < 0.001, **: *p* < 0.01; *: *p* < 0.05, number of sessions indicated in ‘a’).

**Extended Data Fig. 3:**
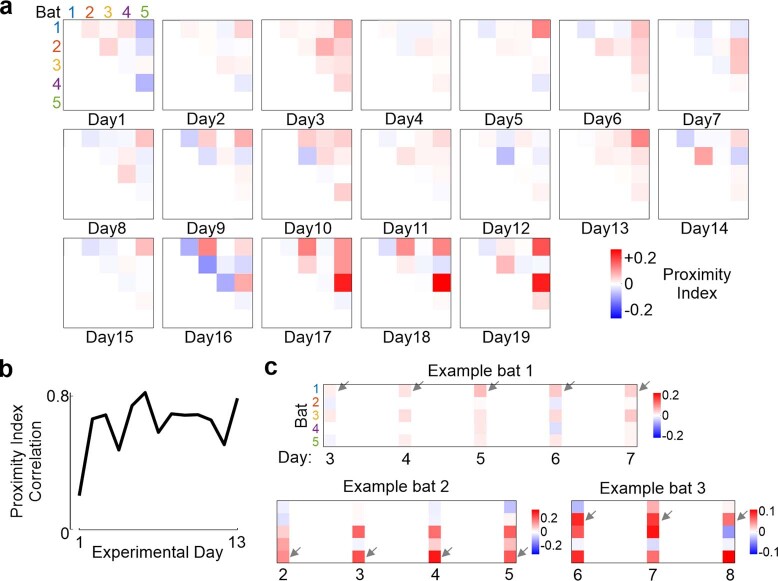
Proximity index stability over days. **a**, Colour-coded maps for the proximity index matrices across days. Each entry of the matrix represents the proximity index between a pair of bats on a given experimental day (Dataset 1, Methods). **b**, Correlation between proximity indexes for all bat pairs on a given day and the mean pairwise proximity index across all other days. Values refer to the same group of five bats and are averaged across three different datasets (Datasets 1,3 and 4; minimal shared days of exposure: 13 days). **c**, Epochs from three example recorded bats, showing the evolution across days of their proximity indexes (columns) relative to other bats (coloured numbers on the left of the first example bat). Arrows indicate cases of stable social preferences (additional stable preferences can be easily identified).

To study collective spatial behaviour in bats under ethologically relevant conditions, we assembled groups of 5–7 individuals and allowed them to fly freely in a large flight room in which either one or multiple food sources were available (Fig. 1a and Methods). To monitor the position of all of the bats, we established a real-time-location system (RTLS) that enabled high spatiotemporal resolution tracking of multiple individuals simultaneously (Extended Data Fig. 1). Bats were highly active, typically flying many dozens of flights in each behavioural session (77 ± 41 flights per hour per bat, mean ± s.d., *n* = 13 different bats across 87 sessions; Extended Data Fig. 2), a substantial fraction of which occurred along repeated trajectories^24,25^ (Fig. 1a, right). Notably, bats spent a modest percentage of time close to (or at) the feeding sites (18 ± 23%, mean ± s.d., *n* = 13 bats across 87 sessions; Extended Data Fig. 2e) and most commonly flew between a handful of self-selected resting sites that were often occupied by other individuals (Fig. 1b). This resulted in a spontaneously emerging, yet highly structured group spatial behaviour whereby specific combinations of individuals, locations and movement patterns characterized the collective dynamics. Indeed, projecting the bat positions onto a state space of all observed group configurations confirmed that a relatively small fraction of possible states comprised most of the occupancy (Fig. 1c,d; fraction of visited states, 7.2 × 10^−3^; range, 10^−9^–10^−2^; *n* = 87 sessions; Methods). Furthermore, this structured behaviour was largely stable across days (mean correlation ± s.d., *C*_self_ = 0.70 ± 0.05, *C*_others_ = 0.37 ± 0.10, *n* = 6 datasets, *P* < 0.05 for all datasets, Wilcoxon signed-rank test; Fig. 1e and Extended Data Fig. 2f) suggesting the emergence of stable social and spatial preferences of individual group members. To further investigate the social structure in the group, we looked at the spatial proximity between specific individuals, a feature that commonly reflects social preferences in bats^20,23,26^. We found that specific bats spent significantly more time close to one another than what would be expected solely by their spatial preferences (Fig. 1f,g (proximity index) and Methods) and that this tendency remained stable across sessions (Extended Data Fig. 3). The resulting structure combined with the robust and reproducible movement patterns of the bats enabled us to assess neural dynamics during a naturalistic and spontaneously emerging spatial behaviour in social groups.

## Coding for the social nature of flights

**Extended Data Fig. 4:**
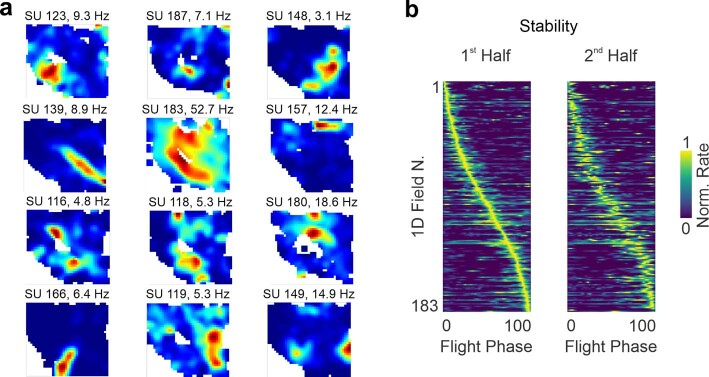
Hippocampal spatial activity in the group context. **a**, Example colour-coded firing rate maps in 2D (x, y). Average firing rate in each x-y spatial location is represented from 0 Hz (dark blue) to the peak rate for the cell (red, noted to the top-right of each map). Total number of spatially informative cells in 2D: 108 out of 147 flight-active cells from three bats (Methods). **b**, Trial-averaged activity from all significant firing fields recorded on flight paths (n = 183 fields from 95 units and three bats; Methods), calculated on random halves of the trials. Activity is rescaled between takeoff (Flight Phase: 0) and landing (Flight Phase: 100). The plot on the left is sorted by the location of peak activity; same sorting is applied to the plot on the right.

We wirelessly recorded the activity of dorsal CA1 and CA2 hippocampal neurons from Egyptian fruit bats engaged in the group behaviour (Fig. 1h,i and Methods). A total of 254 well-isolated single units were recorded over 46 foraging sessions involving groups of 6 or 7 bats (Extended Data Fig. 2). The obtained results were largely consistent across both hippocampal subregions (Extended Data Table 1) and are therefore described together. Most recorded units in both regions carried significant spatial information about the recorded bat’s position (73%, 108 out of 147 flight-active cells when assessed in 2D and 72%, 95 out of 132 flight-active cells along specific trajectories; Fig. 1j,k and Extended Data Fig. 4a), activating in one or multiple locations in the room and, most commonly, around take-off or landing spots (Fig. 1j,k). The resulting spatial firing patterns were largely stable throughout the session (mean Spearman correlation ± s.d., 0.73 ± 0.21, *n* = 183 cells × trajectories; Fig. 1l and Extended Data Fig. 4b). Thus, hippocampal activity during spatial movement in the group setting appeared to be consistent with a stable allocentric representation of self-position^7–9^. Yet, the structured activity could also reflect the regular spatial behaviour of the group (Fig. 1a–e), leaving open the possibility that dynamic changes in social configuration could influence neural activity. We therefore took advantage of the reproducibility of aerial movement in this species^25^, along with the natural richness of the group social setting, to examine whether social factors of the group behaviour influenced hippocampal activity.

**Fig. 2:**
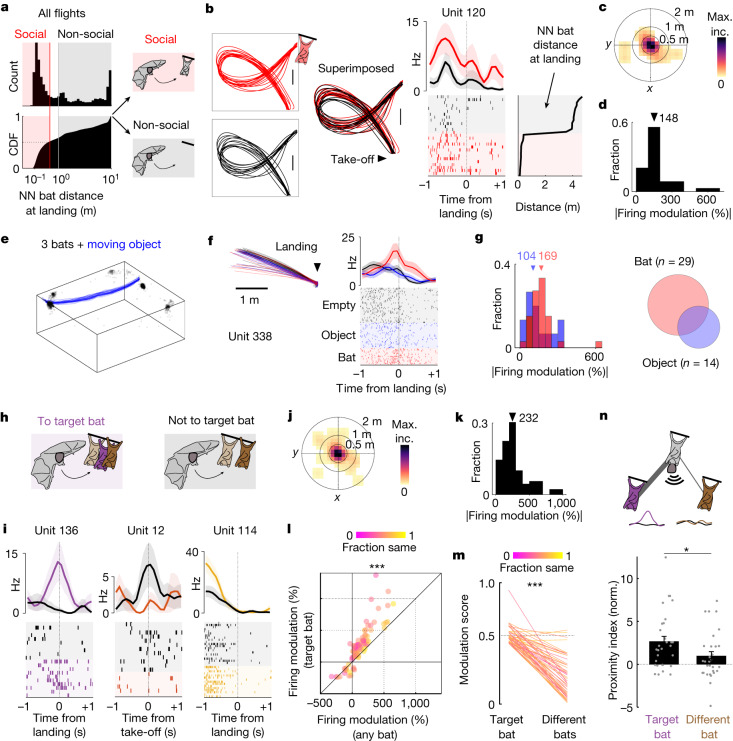
Hippocampal activity is modulated by the social nature of flights. **a**, Left, histogram and cumulative distribution (CDF) of the distance from the nearest-neighbour (NN) bat at landing. Right, flights are divided into social (red area) and non-social (grey area) on the basis of the nearest-neighbour bat distance (Methods). **b**, An example unit modulated by the social nature of a flight. Left, top view of flight paths during social (red, bat at the landing spot) and non-social (black) flights. Note that trajectories for social and non-social flights are highly overlapping. Right, the average firing rate (top) and raster plot (bottom) during social (red) versus non-social (black) flights. The shaded areas indicate the s.e.m. Flights are sorted by nearest-neighbour bat distance at landing (right). Scale bars, 1 m. **c**, 2D heat map of positions relative to take-off or landing locations when social modulation of firing rates occurred (normalized to maximum incidence (max. inc.); *n* = 34 cells × location; 10 around take-off, 24 around landing, 31 cells from 3 bats). **d**, The distribution of firing modulation relative to the baseline (the same cells and locations as in **c**). **e**, Landing positions from all bats (randomly subsampled for visualization; black) and average trajectories of the object (blue) for all sessions. Note that the object (which was controlled from outside the room) moves between common landing locations for the bats. **f**, An example unit modulated by the presence of a bat at the landing location, but not by the object. Left, top view of landing trajectories during flights landing on an empty location (black), close to an object (blue) or to a bat (red). Note that trajectories are highly overlapping. Right, the average firing rate (top) and raster plot (bottom) during the flight types described above. The shaded areas indicate the s.e.m. **g**, Left, the distribution of firing modulation relative to the baseline for neurons modulated by an object (blue, *n* = 15 cells × location, 14 cells from 2 bats) or a bat (red, *n* = 33 cells × location, 29 cells from 2 bats). The triangles indicate the respective median values. Right, summary of the numbers of cells that were responsive to a bat (red) and/or to an object (blue). Responses to the bat were significantly different compared with responses to the object (*P* < 0.05; Methods) for the large majority of both cell classes (84% bat modulated, 71% object modulated). **h**, Schematic of flights to a specific target bat (left; purple bat) and flights not to the target bat (right; the target bat is absent). **i**, Three representative units showing modulation of firing, around take-off or landing, for flights to a target bat that was present (colour) or absent (grey). The average firing modulation (top; shaded area is s.e.m.) and raster plots (bottom) are shown. **j**,**k**, Positions relative to take-off or landing locations (**j**) and the distribution of firing modulation relative to the baseline (**k**) as described in **c** and **d**, respectively, but for cells modulated by a specific target bat (*n* = 89 cells × location × target bat; 47 around take-off, 42 around landing, 58 cells from 3 bats). **l**, Modulation for flights to a specific target bat (vertical axis) versus flight to any bat (equivalent to social versus non-social; horizontal axis). Each marker represents a triplet (cell × location × target bat, *n* = 88 triplets, 58 cells from 3 bats). The marker colour indicates the fraction of flights that share the same class (to the target or not to the target, or to any or to none). Note that modulation is significantly higher (above the unity line) for specific bats compared with the presence or absence of any bat (*P* = 5 × 10^−14^, Wilcoxon signed-rank test). **m**, Modulation scores (Methods) for flights to a specific target bat versus flights to different target bats. The line colour indicates the fraction of flights that share the same class (to the target, not to the target) when considering the target bat and a different bat (*n* = 56 pairs, 19 cells from 3 bats; *P* = 9.5 × 10^−11^, Wilcoxon signed-rank test). **n**, Top, schematic of social proximity between the recorded bat (grey) and either the target bat (purple) or a different bat (brown). Bottom, the normalized proximity index (bars represent the average, markers show single values; Methods) between the recorded bat and either the target or a different bat (*n* = 30 cells from 3 bats; *P* = 0.03, Wilcoxon signed-rank test). The error bars represent the s.e.m. *P < 0.05, ***P < 0.001. Source Data

**Extended Data Fig. 5:**
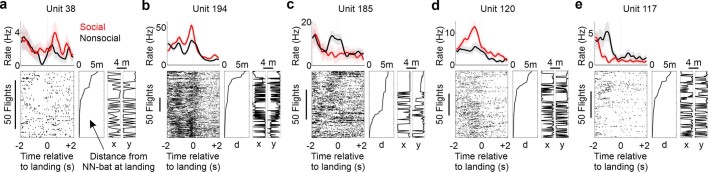
Example socially modulated neurons around landing. Example cells whose firing is modulated by the social nature of a flight around landing time. The proximity of another bat to the landing location (red firing profile and lower part of the raster plots) can correspond to both an increase (**a**, **b** and **d**) or a decrease (**c** and **e**) in the firing rate of the recorded cells. This modulation is not associated to systematic differences in landing positions (note the fairly random profiles of x and y coordinates at landing). **a-e**, For each unit, the top part of the panel shows the average firing rate around landing for social (red) vs. nonsocial (black) flights. Shaded areas represent SEM. The bottom part of the panel displays the spike raster plot around landing, the distance from nearest-neighbour bat (NN-bat) and the x, y coordinates at landing for all the flights executed during the session, sorted by the NN-bat distance.

**Extended Data Fig. 6:**
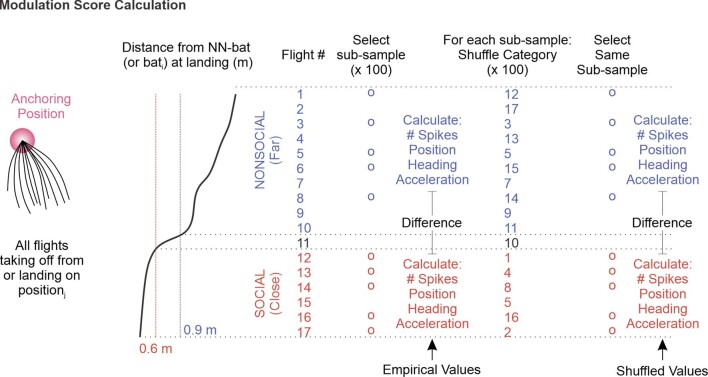
Modulation scores calculation. Description of the procedure for the calculation of the modulation scores (Methods). Scores were calculated for a given neuron and anchoring location (takeoff or landing, pink circle on the left, position_j_) with enough flights (Methods). Scores were calculated for two cases: *social* vs. *nonsocial flights*, by considering the nearest-neighbour bat distance at landing (NN-bat) or *flights to target bat* vs. *not to target bat*, by considering the distance from a specific bat at landing (bat_i_). Here we describe the procedure for social vs. nonsocial flights (a similar method was applied for *flights to target bat* vs. *not to target bat*, Methods). Flights were sorted based on the NN-bat distance at landing and classified as social (landing close to a bat, red) or nonsocial (landing far from a bat, blue). A subsample of five social vs. five nonsocial flights was extracted at random – to balance the number of social vs. nonsocial trials - and firing frequency, position, heading and acceleration in the time bin of interest were calculated. The differences between average firing frequency, position, heading and acceleration for social vs. nonsocial flights were tested for significance by comparing their empirical values against a shuffled distribution obtained by randomly permuting the category of a flight (social vs. nonsocial) 100 times and recalculating the same differences on the considered subsample of flights. This procedure was repeated 100 times, across different random subsamples of five social vs. five nonsocial flights, thus obtaining 100 p-values for differences in the variables of interest. Modulation scores for each of the four variables of interest (firing, position, heading and acceleration) were calculated as the fraction of subsamples with significant (*p* < 0.05) differences in the associated variable between social and nonsocial flights.

**Extended Data Fig. 7:**
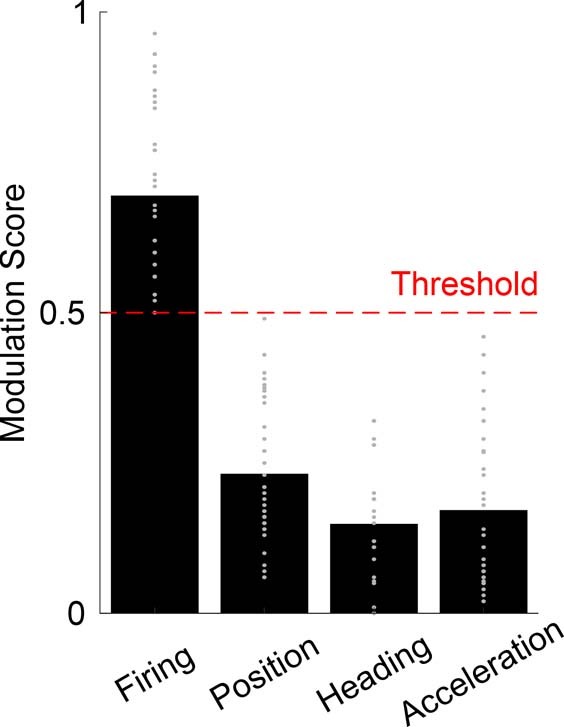
Modulation scores for socially modulated neurons. Modulation scores for differences in firing, position, heading and acceleration for the socially modulated neurons (n = 34 cells x locations; 10 around takeoff, 24 around landing, 31 cells from three bats). The threshold (red dashed line) indicates the minimal firing score and the maximal positional and kinematic scores, to be classified as a socially modulated neuron under this conservative exclusion criteria (Methods). Bars represent the average, markers represent single values.

A key decision any social animal needs to make is whether to move towards a location that is presently vacant or occupied by other individuals. This feature cannot be assessed in dyadic, reward-driven behaviours where, as a consequence of training and task configuration, a given animal is either always present or absent in a fixed location^12,13^. By contrast, in the unconstrained group settings, bats spontaneously flew to multiple locations in which other individuals were either present or absent. Advantageously, when computing the distribution of distances from nearest-neighbour bats at landing (Fig. 2a), we observed a natural subdivision: about half of the flights (51% of total *n* = 57,806 flights across 13 different bats and 87 sessions) landed close to (or on) another bat (termed social flights), whereas the remaining flights landed far from any bat (termed non-social flights). Examining the activity of single units for social versus non-social flights revealed that many cells showed substantial changes in firing between the two conditions without any systematic change in spatial behaviour (Fig. 2b and Extended Data Fig. 5). To evaluate differences in hippocampal firing induced by the social nature of flights while controlling for spatial behaviour, we first tested using a stepwise generalized linear regression model (GLM; Methods) whether including the social versus non-social category could better explain the firing rate around take-off or landing, when most spatially modulated cells were active (Fig. 1k). We found that, for a substantial percentage of cells (89%, 144 out of 162 analysable units), a model including the social nature of a flight performed significantly better compared with either a constant model or a model including the positions around take-off or landing (*P* < 0.05, deviance test; *n* = 162 tested units; Methods). Systematic analysis of firing around specific locations for take-off and landing also resulted in a considerable percentage of cells modulated by the social nature of flights without a significant change in position between social and non-social flights (49%, 66 out of 135 analysable units; Methods). Finally, we considered that hippocampal neurons can be modulated by other kinematic variables, in addition to position^8,9,27^. We therefore took a conservative approach that examined whether we could find cells that are modulated by the social nature of flights yet showed minimal change in relevant kinematic variables, including position. To do so, we calculated four ‘modulation scores’ for each neuron that assessed the extent of significant change in firing, position, heading and acceleration between social and non-social flights (Methods and Extended Data Fig. 6). We found that, even under these stringent exclusion criteria, nearly a quarter of the single units (23%, 31 out of 135 analysable units; Fig. 2c,d) showed significant modulation of firing between social and non-social flights, primarily near the take-off and landing spots (Fig. 2c) and, importantly, with negligible differences in position, heading or acceleration (Extended Data Fig. 7). While representing an underestimation of the percentages of socially modulated cells, these findings nonetheless demonstrate that the social nature of self-selected flight had a profound influence on the activity of many hippocampal neurons, independently of changes in position and other kinematic variables.

**Extended Data Fig. 8:**
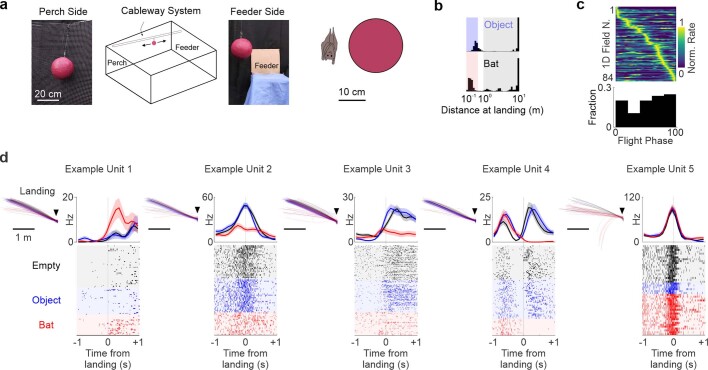
Object experiment: additional quantifications and single unit responses. **a**, Illustration of the experiment involving three bats and an object (red Styrofoam ball, pictures). The object could be moved between locations in the room where bats land for resting (perch) or feeding (feeder). Scale-preserving representation of a typical bat (perching pose) and the object that was used in the experiments is shown on the right. **b**, Histogram of the distances at landing relative to the object (top) or the bats (bottom). Flights are divided into object/bat-present (blue/red area) or absent (grey areas), based on the respective distances (Methods). **c**, *Top*: trial-averaged activity from all significant spatial firing fields recorded during the object-experiment on flight paths (n = 84 fields from 2 bats), rescaled from takeoff to landing and sorted by location of peak activity. *Bottom*: distribution of the fields’ peak location as a function of flight phase (0 is takeoff, 100 is landing) for the same 1D fields shown on top. **d**, Example units recorded during the object experiment. *Left of each example*: Top view of landing trajectories during flights landing in the location (triangle) when it is empty (black), object is present (blue) or bat is present (red). Note that trajectories are highly overlapping. *Right of each example*: average firing rate (top) and raster plot (bottom) during the above flight types. Shaded areas indicate SEM. Note that units can also be largely unaffected by the presence of an object or a bat at the landing location (last example on the right).

We next examined whether similar responses would be observed for any dynamic object that could be present or absent at the landing location. We therefore conducted an additional set of experiments in which a group of three bats foraged freely in the room while an object (a Styrofoam ball) was moved to be either present or absent at the resting and feeding locations (Fig. 2e, Methods and Extended Data Fig. 8a,b). We recorded the activity of 119 hippocampal single units from two bats and, consistent with our previous findings, found both spatial responses (61%, 71 out of 116 flight-active cells along specific trajectories; Extended Data Fig. 8c) and social responses (Fig. 2f and Extended Data Fig. 8d; 28%, 29 out of 103 analysable units using the same conservative criteria as above). Socially modulated cells were responsive to the presence of another bat at the landing spot but were largely unaffected by the presence of an object (Fig. 2f and Extended Data Fig. 8d). Notably, about 16% of the recorded neurons (14 out of 86 analysable units) were modulated by the presence of the object, with changes in firing rate that tended to be smaller than those observed for socially modulated cells (Fig. 2g, left). Yet, we found only a small superposition between the two subpopulations (Fig. 2g, right), suggesting that responses to the presence or absence of a conspecific are largely distinct from those to an object devoid of social value.

## Modulation by identity and social proximity

**Extended Data Fig. 9:**
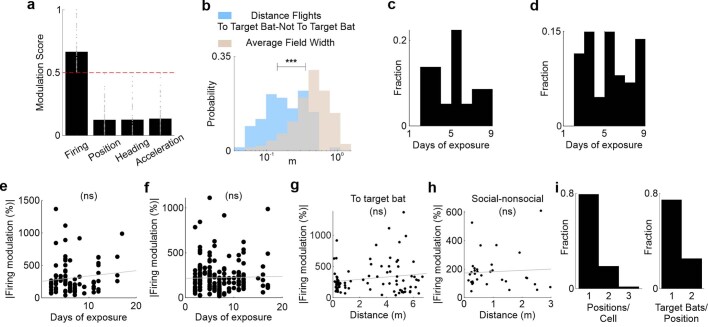
Features of the socially modulated neurons for specific target bats. **a**, Modulation scores for differences in firing, position, heading and acceleration for the socially modulated neurons around flights to specific target bats (n = 89 cells x location x target bat; 47 around takeoff, 42 around landing, 58 cells from three bats). The threshold (red dashed line) indicates the minimal firing score and the maximal positional and kinematic scores, to be classified as a socially modulated neuron under this conservative exclusion criteria (Methods). Bars represent the average, markers represent single values. **b**, Distribution of the average distance between positions (during neural modulation) for *flights to target bat* vs. *flights not to target bat*, for all the socially modulated neurons shown in (a) (light blue), compared with the average field-width calculated for all significant 1D-fields (n = 183 fields from 95 units and three bats, light brown). The average field width is calculated as the average width in x, y, and z (where the firing frequency dropped below the half-maximal frequency at the centre of the field). Note that positional differences between flights for socially modulated cells are much smaller than the typical field size (*p* = 3.8 x 10^-20^, n = 89 pairs of *flights to target bat* vs. *not to target bat*, n = 183 1D-fields, Wilcoxon rank sum test). **c**, Fraction of detected cells modulated by a target bat binned by the number of days of exposure to the experiment (chi-square goodness-of-fit test against a uniform distribution: *p* = 0.0012, n = 45 cells from three bats). **d**, same as in **c**, for cells modulated by a target bat using less stringent criteria not involving the modulation scores (Methods). Incidence of socially modulated cells was significantly different from a uniform distribution (*p* = 0.008, n = 65 cells from three bats, chi-square goodness-of-fit test). **e**, Social modulation for flights to a specific target bat (vertical axis) versus number of days of exposure to the experiment (horizontal axis). Each marker represents a triplet (cell x location x target bat; n = 89 triplets, 58 cells from three bats). Line represents the least-squares interpolating line. ns means non-significant correlation (c = 0.12, *p* = 0.25). **f**, same as in **e**, for cells modulated by a target bat using less stringent criteria (Methods). Pearson’s c = 0.02, p = 0.74, n = 194 cells x location x target bat, 87 cells from three bats. **g**, Social modulation for flights to a specific target bat (vertical axis) versus distance from the target bat at the time of the modulation (horizontal axis). Each marker represents a triplet (cell x location x target bat, n = 89 triplets, 58 cells from three bats). Line represents the least-squares interpolating line (c = 0.17, *p* = 0.1). Correlation was not significant also when separately considering cells modulated around takeoff or landing (c = -0.13, p = 0.37, n = 47 around takeoff, c = −0.15, p = 0.34, n = 42 around landing). **h**, Same as in g, but for modulated cells during social vs. nonsocial flights (n = 34 cells x location, 31 cells from three bats; c = 0.06, *p* = 0.73). **i**, Distributions of the number of positions per neuron and number of target bats per position, for all the socially modulated neurons included in (a). Note that most of the cells are socially modulated around one single location and for a single target bat. ns indicates non-significant correlation.

**Extended Data Fig. 10:**
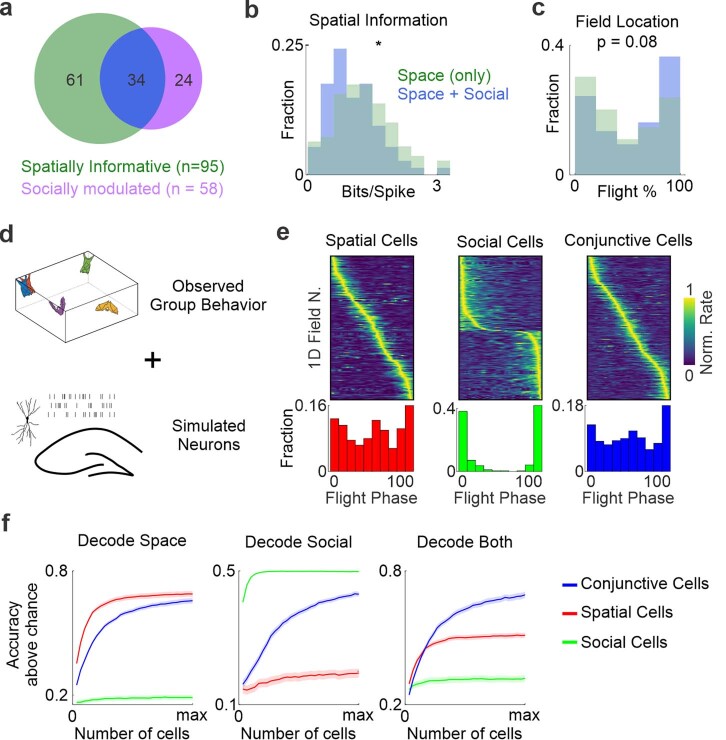
Interplay between spatial and social responses in empirical and simulated data. **a**, Venn diagram summarizing the numbers of cells that were spatially informative on flight paths (green) and/or socially modulated by a target bat (magenta). **b**, Distribution of spatial information for different cell classes (blue vs green in **a**), *p* = 0.012, Wilcoxon rank sum-test. **c**, Distribution of the fields’ peak location as a function of flight phase (0 is takeoff, 100 is landing) for the same cell classes shown in **b**. **d**, *Top*: schematic description of the simulations, generated by combining observed group behaviour (top) with simulated neurons (bottom). **e**, *Top*: trial-averaged activity from all significant spatial firing fields generated by simulating three different neuronal types (Methods), rescaled from takeoff to landing and sorted by location of peak activity. *Bottom*: distribution of the fields’ peak location as a function of flight phase (0 is takeoff, 100 is landing) for the same 1D fields shown on top. **f**, Accuracy above chance for decoding the landing location (left), the social nature of a flight (centre) or both (right), using activity from different simulated cell classes, as a function of the number of neurons (max corresponds to ~30 neurons for all cell classes, methods).

Having observed the impact of social movement on hippocampal activity, we next examined what features of the collective social setting could contribute to the observed neural modulation. To date, social and positional modulation of hippocampal neurons have largely been examined in the context of either dyadic interactions, for which identity selectivity could not be assessed^12,13,16,24^, or physically constrained interactions^17,18,28–30^, lacking the dynamic aspect of self and others’ spatial movement. Both aspects—identity and spatial movement—were inherently embedded in the group setting and enabled us to examine whether and how identity information is represented in an animal that is actively navigating through a social space. We again took advantage of the structured nature of the bat group behaviour, whereby different animals shared similar spatial locations at different times, in turn enabling us to distinguish between the general presence of any bat at a given location and that of a specific bat in the same location. We found that many neurons showed robust modulation of firing on the basis of the presence of a specific bat at the landing spot (termed the target bat; 61%, or 87 out of 142 analysable units modulated by the presence of a specific target bat without a significant change in position; Fig. 2h,i and Methods). To further assess the significance of this effect, while again excluding potential changes in spatial behaviour, we used the same conservative approach as before and calculated the modulation scores associated to differences in firing, position, heading and acceleration (Methods). We found that a substantial fraction of the hippocampal neurons (41%, 58 out of 142) showed large firing modulation when flying to the target bat, centred around the take-off and landing locations (Fig. 2j,k) and, importantly, low positional, heading or acceleration changes (Extended Data Fig. 9a,b). The identity-modulated neurons were present as early as 2 days after exposure to the collective environment (Extended Data Fig. 9c,d) and the magnitude of social modulation did not significantly correlate with days of exposure (Extended Data Fig. 9e,f). Furthermore, the observed modulation was independent of the time in the session at which flights towards specific locations and group configurations occurred (Methods) as well as of the distance from the target bat (Extended Data Fig. 9g,h). Most units showed modulation when taking off from or landing at one single position in the room and for one single target bat (Extended Data Fig. 9i), suggesting a conjunctive code for space and identity. In support of the tight interaction between spatial and social signals in the hippocampal neurons, we found that many of the cells modulated by a target bat were also spatially informative (Extended Data Fig. 10a) despite carrying significantly less spatial information than non-socially modulated cells and having spatial field locations that were more skewed towards the end of flight (Extended Data Fig. 10b,c). Computational simulations of different functional cell classes (Methods and Extended Data Fig. 10d,e) suggest that such conjunctive representations can be advantageous for simultaneously decoding spatial and social aspects of behaviour in collective settings (Extended Data Fig. 10f).

**Extended Data Fig. 11:**
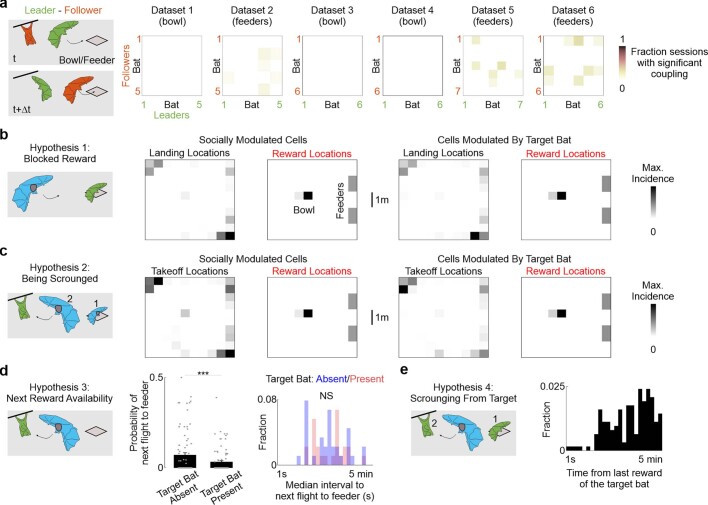
Examining alternative reward-related explanations for the social modulation. **a**, *Left*: Schematic description of leader-follower dynamics, where flights to food reward of one bat are preceded or followed by similar flights of another bat at a brief time interval. *Right*: Incidence of significantly coupled pairs of bats across datasets (Methods). **b**, *Left*: Schematic description of the first reward-related alternative explanation: neural responses are caused by reward being blocked by another bat. *Right*: 2D-distribution of landing locations where cells were socially modulated (or modulated by a specific target bat) vs. reward locations in the same sessions. Note that there is little overlap between the two. **c**, *Left*: Schematic description of the second reward-related alternative explanation: neural responses are associated with opportunistic behaviour of other bats, after the recorded bat came back from reward locations. *Right*: 2D-distribution of takeoff locations preceding locations where cells were socially modulated (or modulated by a specific target bat) vs. reward locations in the same sessions. Note the little overlap between the two. **d**, *Left*: Schematic description of the third reward-related alternative explanation: neural responses reflect availability of reward at the next flight, according to the presence (reward available) or absence (reward potentially blocked) of a specific bat at the landing spot. *Middle*: Probability of the next flight to reward, depending on presence or absence of the target bat at the landing spot (for cells modulated by a specific target bat, n = 89 cells x location x target bat; 58 cells from three bats, *p* = 1.5 x10^-4^, Wilcoxon signed rank test). Error bars represent SEM. *Right*: Median interval between landing and the takeoff of next flight to reward for the same cells, depending on the presence or absence of the target bat at the landing spot (n = 15 intervals from 11 cells, three bats, *p* = 0.73, Wilcoxon rank sum test). **e**, *Left*: Schematic description of the fourth reward-related alternative explanation: neural responses reflect an opportunity to scrounge some reward from the target bat. *Right*: Median interval between the time of the neural response and the last reward of the target bat. Note that most rewards are collected minutes before the neural response. Number of socially modulated cells or cells modulated by a specific target bat are reported in the main text. NS: *p* > 0.05, ***: *p* < 0.001.

To further assess the specificity of the response to the target bat, we reasoned that, if modulation in firing was not caused by the presence or absence of a specific bat, then we should obtain similar effects when partitioning the flights into social (any bat) versus non-social (no bats). However, we found that the identity-specific modulation was systematically higher than when considering social versus non-social flights (Fig. 2l; *P* = 5 × 10^−^^14^, Wilcoxon signed-rank test, *z* = 7.54, *n* = 83 cells × location × target bat). Moreover, for all of the cells modulated by a specific target bat, we quantified the modulation that the same cell would show when partitioning flights according to identities of different bats. We found that most units modulated by a specific bat were far less modulated by others (Fig. 2m; *P* = 9.5 × 10^−11^, Wilcoxon signed-rank test, *z* = 6.47, *n* = 56 cells × location × target bat). Finally, using the observed social structure in the bat group (Fig. 1f,g), we found that the target bats (associated with significant firing modulation) were more likely to be socially proximal to the recorded bat (Fig. 2n, *P* = 0.03, Wilcoxon signed-rank test, *z* = 2.15, *n* = 30 cells from three bats), therefore suggesting a relationship between the social proximity and the differential hippocampal response. Finally, although coordination or competition for reward were not explicitly instructed in our experiments, we tested whether specific interactions between reward and identity^31^ could account for the social responses that we observed. We found little to no evidence of leading–following behavioural dynamics around reward (Extended Data Fig. 11a) and that neural modulation was mostly incompatible with several reward-based hypotheses (Extended Data Fig. 11b–e). Furthermore, only a minority of the recorded cells showed a significant response to the presence or absence of a reward (19%, 14 out of 73 units; and 20%, 3 out of 15 units modulated by a bat; Methods). Indeed, even when removing all of the flights to and from reward locations, there was almost no change in the fraction of socially or identity modulated cells (minimal decrease from 23% to 22% for social–non-social modulation and from 41% to 38% for cells modulated by target bat). These results suggest that the socially modulated and identity-specific neural responses that we observed were largely independent of reward-driven behaviour.

## Neural responses to others’ behaviour

**Fig. 3:**
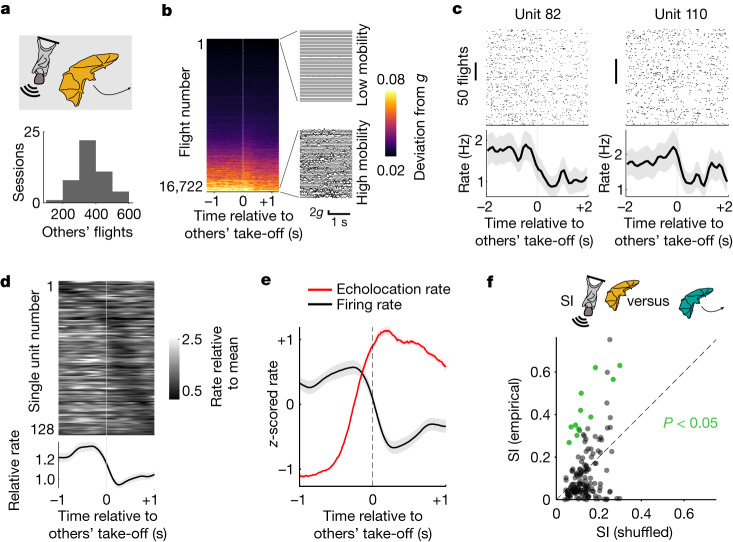
During rest, hippocampal neurons are sensitive to others’ behaviour but not identity. **a**, Top, schematic of the flights of other bats while the recorded bat is not moving. Bottom, the number of flights from other bats during the recorded sessions. **b**, Accelerometer signal (absolute deviation from *g*; colour bar; Methods) recorded from the implanted bats around take-off of other bats. Fifty traces corresponding to the raw accelerometer signal during low-mobility trials (top inset) or high-mobility trials (bottom inset) are shown on the right. **c**, Two representative units that were significantly modulated around the take-off of other bats. Top, raster plots. Bottom, the average firing rate. The shaded areas indicate the s.e.m. Note the decrease in firing around the take-off of other bats. **d**, The trial-averaged firing rate for all units that were significantly modulated around the take-off of other bats (*n* = 128 cells from 3 bats), sorted by the time of significant modulation. The average across all cells is shown below. The shaded area indicates the s.e.m. **e**, The average firing rate in stationary bats and echolocation rate (*z*-scored, median number of take-off events per cell was 118) around the take-off of other bats (*n* = 84 cells from 3 bats). The shaded area indicates the s.e.m. **f**, The selectivity index (SI) of hippocampal responses during flights of specific bats: empirical (vertical axis) versus shuffled (horizontal axis) data. A selectivity index was calculated for each resting position of the recorded bat (*n* = 149 cells × position from 113 cells, 3 bats). Significant values (Methods) are shown in green. Source Data

**Extended Data Fig. 12:**
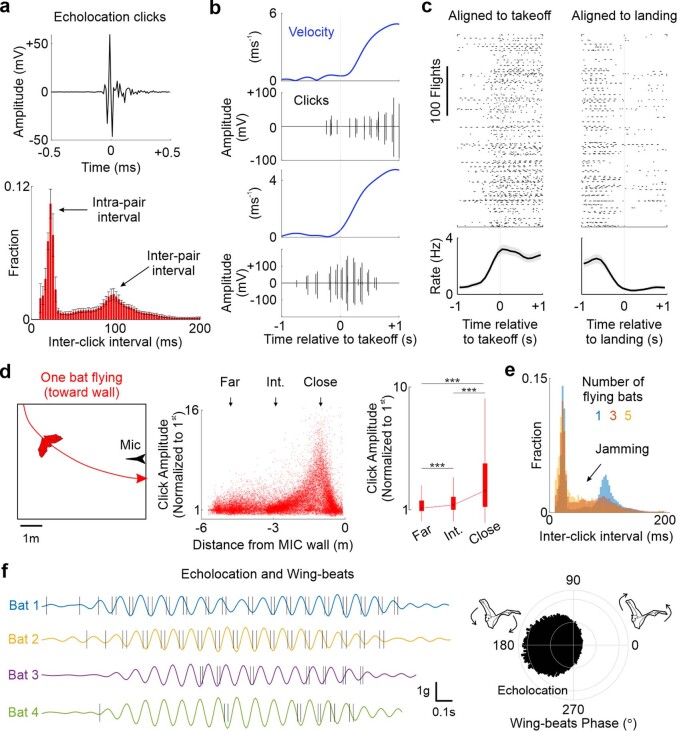
Increase of echolocation emitted by the flying bats around flight time during group foraging. **a**, *Top:* average waveform for all echolocation clicks detected in one representative session (n = 7294 clicks from a session involving six bats). *Bottom:* distribution of inter-click-intervals detected over all sessions involving sound recordings. Each bar represents the mean fraction of inter-click-intervals at the corresponding time bin, averaged across sessions (n = 33 sessions involving 5-7 bats, 3115-18452 clicks per session). Error bars represent s.d. Note the bimodal nature of the distribution, as expected from the temporal features of echolocation from single bats, where clicks are typically emitted in pairs (intra-pair interval ~ 20 ms) separated by ~100 ms (inter-pair interval). **b**, Velocity profiles (blue) and detected clicks (black) around takeoff from two example flights (same session as reported in ‘a’). **c**, Raster plots aligned to takeoff (left) and landing (right) showing detected echolocation clicks around flight times (same session as in ‘a’, 389 flights from six bats). The average echolocation rate is shown below the raster. Shaded areas indicate SEM. Note the sharp rise in echolocation rate at takeoff. **d**, *Left*: Schematic of the examined flights, where one single bat is flying towards the side of the room where the recording microphone (MIC) is located. *Centre*: Amplitude of all detected echolocation pulses for the examined flights (n = 25671 echolocation pulses from 1397 flights), normalized to the first one in the flight, as a function of the distance of the flying bat from the wall where the microphone is located. Distance classes (far, intermediate, and close, obtained by partitioning the distance range into three equal intervals) are indicated on top. *Right*: Box plots (lines indicate max and min values, rectangle indicates 25^th^-75^th^ percentiles and dots indicate median) for the normalized click amplitude at the distances indicated in the middle panel (n = 5731, 8638 and 11300 echolocation pulses at far, intermediate and close, respectively; Kruskal-Wallis test with Tukey multiple comparison test, *** represents significance levels of multiple comparisons: *p* = 0 for all pairs). **e**, Histogram of the inter-click intervals for flights where one, three or five bats (maximum) were flying at the same time. Jamming indicates an increase in the clicks separated by less than 100 ms and more than 20 ms. **f**, Relationship between echolocation and the wing-beat cycle. *Left*: examples from four different bats showing the accelerometer signal during flight and the timing of detected echolocation pulses (vertical lines). Note that click pairs tend to occur around the downstroke. *Right*: Distribution of click phase relative to the wing-beat cycle for all detected clicks (86,099) when one bat is flying.

**Extended Data Fig. 13:**
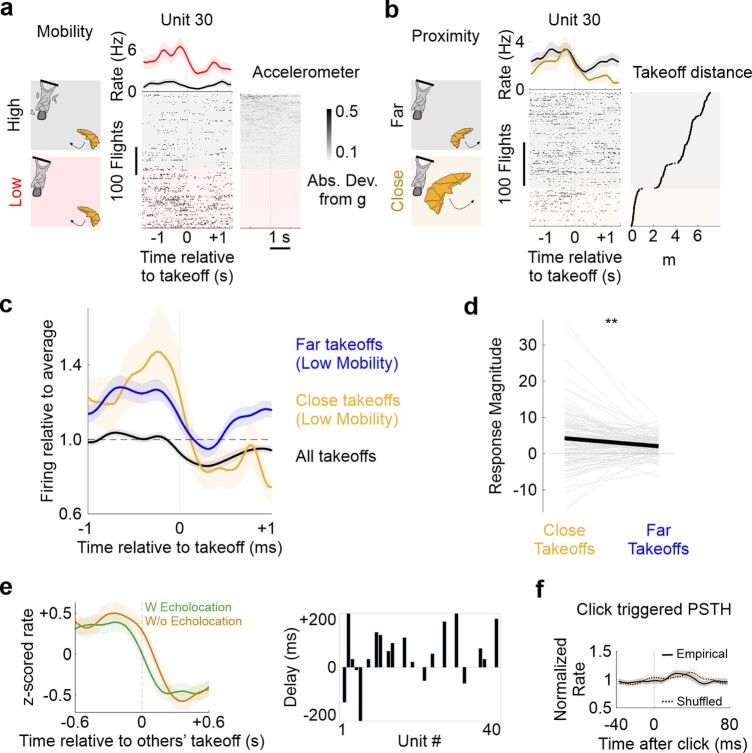
Additional analyses related to hippocampal responses in stationary bats around others’ takeoff. **a**, **b**, Activity from an example neuron (same on both panels) during stationary epochs of the recorded bat and aligned to takeoff of other bats. Trials in **a** are sorted based on the overall mobility of the recorded bat (Methods). Trials in **b** are sorted by the distance of the recorded bat from the bat that was taking off. For both panels: schematic representation (left), raster plot of all spikes and average firing frequency (centre) across conditions (red vs. black: low mobility vs. high mobility; yellow vs. black: close vs. far takeoff events). Shaded areas represent SEM. Plot on the right of panel **a:** accelerometer signal (absolute deviation from g) recorded from the stationary bat around takeoff of the other bats. Plot on the right of panel **b:** takeoff distance of the other bats from the recorded bat. **c**, Firing rate relative to baseline for the same cells (n = 106 from three bats) averaged across three different conditions: takeoff events happening close (yellow, < 1 m) to the recorded bat during low mobility epochs, same but for far takeoffs (blue, > 1 m) and for all takeoffs (no mobility or distance constrain) for comparison (black). Activity is aligned to takeoff and shaded areas represent SEM. **d**, Magnitude of the social modulation (Methods) around others’ flights for the same cells during low mobility-close takeoffs and low mobility-far takeoffs (*p* = 0.006, Wilcoxon sign rank test, 106 cells from three bats). **e**, *Left*: Average firing rate in stationary bats (z-scored) for the same cells (n = 75 cells from three bats) around takeoff of other bats on trials with detected echolocation (green) or without detected echolocation (brown). Shaded areas indicate SEM. *Right*: Delays between responses with or without echolocation for the subset of units (n = 39) fulfilling criteria for accurate delay estimation (Methods). **f**, Average firing rate (normalized to baseline) around echolocation pulses emitted when no bats are flying (n = 84 cells from three bats, modulated around takeoff of other bats) vs. shuffled rate (dotted line), calculated by randomly sampling from the same epochs. Shaded areas indicate SEM.

We considered the fact that, in a natural group setting, the most common spatial state of any given individual is relative stationarity, that is, remaining in the same spatial location while others are moving. Yet, in a dynamic social context, it is important to constantly monitor the behaviour of other group members that might be changing positions. Indeed, we observed that a bat’s stationary state was constantly interleaved by hundreds of other bats flights (Fig. 3a and Extended Data Fig. 2b). These flights were often accompanied by a sharp increase in echolocation rates produced largely by the flying bats around take-off or landing (Extended Data Fig. 12)—a highly relevant sensory signal in the umwelt of bats^19,32^. We therefore took advantage of the high number of flights and temporal precision of the echolocation signal to explicitly examine the existence of hippocampal responses to the spatial movement of other group members and their broadcasted sensory signals. To exclude contact-induced responses and control for movement-related firing variance, we monitored the bat’s movement using an onboard accelerometer and restricted our analysis to epochs of low mobility of the recorded bat (Fig. 3b and Methods). We found that most single units were significantly modulated around the take-off of other bats (128 out of 177 units with enough low-mobility take-off events, 72%; Fig. 3c,d and Methods). The most common response profile was a transient suppression of firing after the take-off of other bats (Fig. 3d). This response showed a small but significant dependence on the distance between the recorded bat and the bat that was taking off (*P* = 0.006, Wilcoxon signed-rank test, *z* = 2.75, *n* = 106 units; Extended Data Fig. 13), yet was evident even for distant take-offs (Extended Data Fig. 13b–d), suggesting that it could be mediated by distal sensory cues, such as audition or vision. Consistent with this notion, we found that the average echolocation rate around take-off of others’ flights was aligned with the average neural response from the stationary recorded bat (Fig. 3e; peak of echolocation rate preceding the trough of firing rate by mean interval ± s.e.m., 126 ± 122 ms, *n* = 84 units with enough flights and recorded echolocation). Although there was a small, albeit significant, correlation between the response magnitude and echolocation rate (*c* = 0.012, *P* = 0.028, *n* = 84 units; generalized linear mixed-effects model), we found that echolocation alone was neither a necessary condition for the hippocampal response around the take-off of other bats (as it was also observed for take-offs in the complete absence of echolocation, albeit shifted in time; Extended Data Fig. 13e), nor was it sufficient to evoke substantial firing changes during periods of rest, when no bats were flying (Extended Data Fig. 13f).

Furthermore, the group setting enabled us to examine whether the observed modulation in hippocampal activity of a stationary bat was dependent on the identity of the flying bat. Considering that the position of the recorded stationary bat could also influence this response, we calculated a selectivity index for three different conditions: selectivity for the identity of the other (moving) bat, selectivity for position of the recorded (stationary) bat and selectivity for the identity of the moving bat when the stationary bat was in a specific position (Methods). In all three cases, selectivity indexes were skewed towards small values (mean selectivity indexes, 0.11, 0.18 and 0.13, respectively; *n* = 124, 110 and 113 units with a sufficient number of events; Methods) and were similar to shuffled indexes, with only a relatively small percentage of cells showing significant selectivity (8%, 8% and 10% selective for position, bat and bat given position, respectively, *P* < 0.05, empirical selectivity indexes versus shuffled; Fig. 3f and Methods). These data suggest that—in contrast to the activity during self-selected motion towards specific bats—the response in stationary bats was carrying relatively low amounts of identity-related information.

## Social representation at the population level

**Fig. 4:**
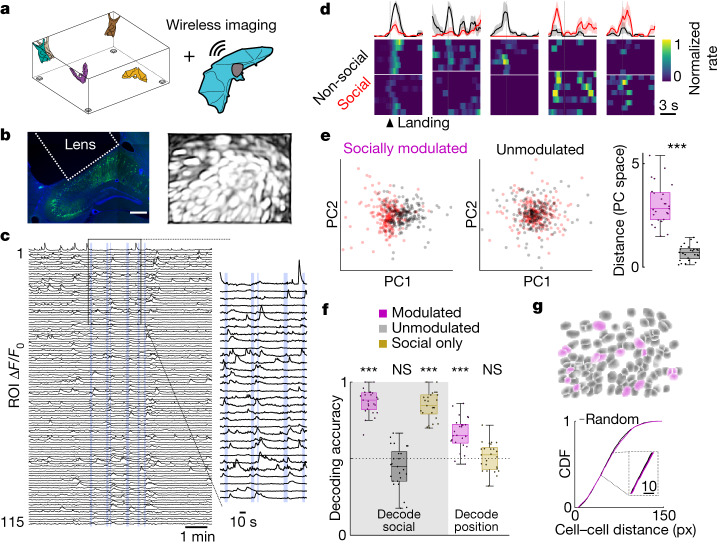
Functional and anatomical organization of social responses at the population level. **a**, Schematic of the experimental paradigm for wireless imaging during collective behaviour. **b**, Left, a coronal section of the dorsal hippocampus from one imaged bat: GRIN lens profile (white dotted lines) and neurons expressing GCaMP6f (green) that were stained for nuclear DAPI (blue). Scale bar, 500 µm. Right, intensity correlation image for one representative FOV showing the imaged cells (bright white). **c**, Fluorescence time series from 115 simultaneously imaged ROIs during group spatial behaviour. Inset, magnification of calcium activity around flights of the imaged bat (blue lines). The cells are from the FOV shown in **b**. **d**, Example activity traces and respective averages for socially modulated ROIs during social (red traces and the bottom portion of the heat maps) or non-social (black traces and top portion of the heat maps) flights around landing. The shaded areas indicate the s.e.m. **e**, Left, each dot represents the first two principal components (PCs) of ensemble activity around single trials of social (red) or non-social (black) landing. All landings are pooled together (Methods), and activity is shown for socially modulated versus unmodulated cells. Right, the distance between centroids (principal component space) for the neural activity around social versus non-social landings for socially modulated (magenta) or unmodulated (grey) cells (*P* = 1.2 × 10^−^^5^, Wilcoxon signed-rank test, *n* = 25 landings from 24 FOVs, 3 bats). **f**, The accuracy in decoding social versus non-social landing (grey area) or the landing position (among the two most common, white area), using activity from different cell ensembles (Methods). Note that, in both cases, chance level is 0.5 (from left to right, *P* = 6.49 × 10^−6^, *P* = 0.97, *P* = 6.48 × 10^−6^, *P* = 1.2 × 10^−5^ and *P* = 0.30, one-sided Wilcoxon signed-rank test; *n* = 25 landings from 24 FOVs, 3 bats). NS, non-significant. **g**, Top, segmented cell profiles for the same FOV shown in **b** (socially modulated cells shown in magenta). Bottom, cumulative distribution function of the pairwise cell distances for socially modulated cells (magenta) and a matched number of randomly sampled neurons (1,000 samples per FOV, black). Inset, a magnified region of the plot. *P* = 0.156, two-sample Kolmogorov–Smirnov test; NS, *P* > 0.05. *n* = 3,548 pairwise distances from *n* = 24 FOVs, 3 bats. px, pixels. The box plots in **e** and **f** show the maximum and minimum values (whiskers), median (centre line) and the 25th to 75th percentiles (box limits). Source Data

**Extended Data Fig. 14:**
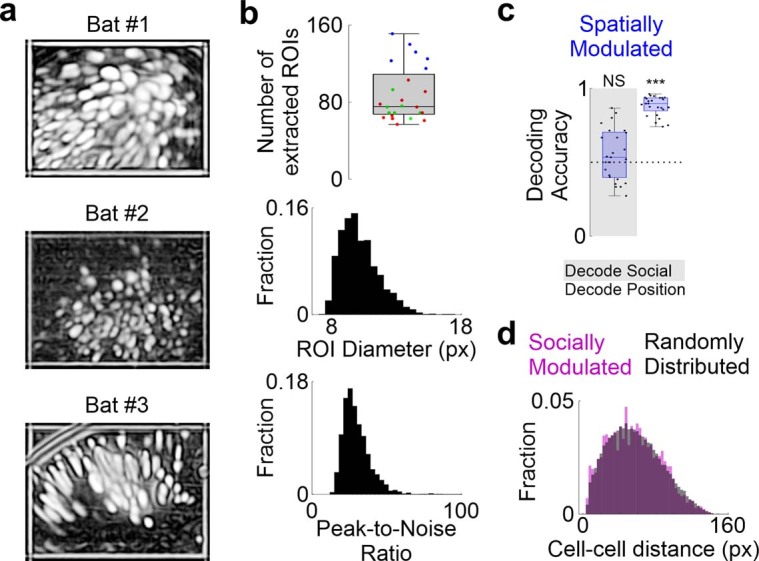
Additional data related to wireless imaging during collective foraging. **a**, Representative FOVs from three imaged bats (intensity correlation images). Bright square borders result from motion correction; bright circular borders correspond to the edges of the GRIN lens. **b**, *Top*: Number of extracted ROIs for the imaged FOVs across three bats (different colours). *Centre* and *Bottom*: distribution of the cell diameters (2 µm *per* pixel) and the peak-to-noise ratios for the extracted ROIs (n = 2109 ROIs from 24 FOVs across three bats). **c**, Decoding accuracy for social-nonsocial landing (grey area) or landing position (among the two most common) from the activity of spatially modulated cells. Note that in both cases, the chance level is 0.5 (n=25 landings from 24 FOVs across three bats, *p* = 0.1 for social, *p* = 7x10^−6^ for position, one-sided Wilcoxon signed rank test). **d**, Distribution of the pairwise cell distances for socially modulated cells (magenta) and a matched number of randomly sampled neurons (1000 samples per FOV, black). The two distributions are not significantly different (two-sample Kolmogorov-Smirnov test, *p* = 0.156, n = 24 FOVs from three bats). Box plots in **b** and **c** report max and min values (vertical lines), median (horizontal line) and 25th-75th percentiles (rectangle).

The results presented thus far addressed the responses of single hippocampal neurons to relevant spatial and social aspects of collective spatial behaviour. Finally, we aimed to explore whether social responses were also evident at the population level and, if so, whether these were anatomically segregated or dispersed. To do so, we used wireless calcium imaging^25^ to record neural activity from flying bats engaged in the same group collective behaviour (Fig. 4a and Methods). We recorded the activity of several dozens of neurons (or regions of interest (ROIs); range, 57–151) per field of view (FOV), expressing the calcium indicator GCaMP6f in the dorsal hippocampus of three bats (Fig. 4b and Extended Data Fig. 14a,b). In agreement with our results from electrophysiological recordings, we found that cells were predominantly active around flight times (Fig. 4c) and that a subpopulation of those changed their activity according to the social nature of flights (mean = 17% across 24 FOVs from three bats; Fig. 4d and Methods), with no significant changes in spatial behaviour (Methods). At the population level, socially modulated cells, but not the socially unmodulated cells, showed distinct ensemble activity for social versus non-social flights resulting in a robust separation in the neural activity space (Fig. 4e; *P* = 1.2 × 10^−^^5^, *n* = 25 landings from 24 FOVs, three bats). Furthermore, we found that even a relatively small number of simultaneously recorded and socially modulated cells (mean, 15) could be used to decode the presence or absence of a conspecific at the landing spot with very high accuracy (around 90%; Fig. 4f (magenta on grey background) and Methods). This was not the case for a matched number of unmodulated cells for which the decoding accuracy was at chance level (black on grey background). Notably, the activity from the same cells could be used to decode the landing position of the recorded bat (Methods), albeit with lesser accuracy (around 70%; Fig. 4f (magenta on white background)), suggesting that social and spatial information may co-exist in the same cells, consistent with the electrophysiological recordings. As expected, the accuracy in spatial decoding decreased to chance levels when we removed the socially and spatially modulated cells (Fig. 4f (brown on white background) and Extended Data Fig. 14c), whereas social decoding remained almost unaffected (brown on grey background). Finally, we investigated whether socially modulated cells showed any anatomical clustering within the imaged FOV (Fig. 4g). When comparing the pairwise-distance distribution between socially modulated cells and that of randomly chosen neurons in the same FOV, we found no significant differences, both when pooling all distances together (*n* = 24 FOVs from three bats, *P* = 0.16; Fig. 4g and Extended Data Fig. 14d) and when individually testing each FOV (only 3 out of 24 FOVs with *P* < 0.05 for socially modulated versus randomly chosen). Combined, these results suggest that both spatial and social information co-exist in the hippocampal population activity and that socially modulated neurons are anatomically dispersed.

## Discussion

Here we took a neuroethological approach that leveraged the natural behaviour of Egyptian fruit bats—a species that regularly negotiates spaces in complex social environments. We allowed groups of bats to behave freely and were guided by the emergent properties of their spatial dynamics. We found a high degree of structure in the group behaviour that was anchored to self-selected locations, movement patterns and social preferences. Using methodologies for wireless neural recordings and simultaneous tracking of multiple freely flying bats, we assessed how hippocampal activity relates to spatial behaviour in dynamic and ethologically relevant group settings.

Advantageously, the emerging behavioural structure contained sufficient variability to dissociate spatial from social components. Bats naturally flew to different locations that were either vacant or occupied by conspecifics, often independently of food reward. This enabled us to assess the unconstrained social nature of spatial behaviour, which could not be addressed in tasks in which the presence (or absence) of another animal was predetermined or experimentally shaped by association with reward^12,13^. Although studies of the hippocampus have consistently reported robust spatial selectivity^7,8^, reports of social responses were much more variable^10,11,16,18^, potentially due to differences in task design, degree of social involvement or other experimental constraints (such as physical confinement of the social stimuli). By allowing a highly social species to engage in an ethologically relevant group behaviour, we found that many neurons robustly modulated their activity during spatial movement towards conspecifics, but less so to objects, even when controlling for positional and other kinematic variables. This representation extended from single cells to populations of neurons and was sufficient to robustly decode both social and spatial aspects of behaviour in collective environments. Furthermore, by considering groups of bats, we found that the socially induced neural modulation was dependent on the identity of individuals^28–30^ and, importantly, could link this selectivity to ongoing spatial movement and locations—a key aspect of spatial behaviour in a social setting.

Notably, although we observed robust identity selectivity during self-movement, we found the opposite during the movement of others. This dissociation may relate to the ecology and sensory signalling of the species. Egyptian fruit bats typically live in dense, dark and often noisy environments in which fine sensory signatures of other individuals can be less discriminable^33^. Moreover, the form of tongue-based echolocation^32^, as compared to laryngeal in most other bat species, probably contains less individual signature in its acoustic structure. Yet, as echolocation is strongly tied to movement in bats, its production can signal the spatial behaviour of other group members. Indeed, we found that echolocation was tightly associated with—but not necessary for—the neural responses in the hippocampus of a stationary bat, which complements reports that self-echolocation may modulate hippocampal activity^34^. Combined, the bat presents an attractive model for studying how the hippocampus, and its associated circuits, encodes incoming streams of sensory information in spatial or social settings by both self and others^35^.

The approach taken here presents an opportunity for understanding fundamental features of collective spatial behaviours across species through comparative work. Even within the order Chiroptera, different species of bats navigate in very different settings, both spatially and socially, requiring selective refinement of sensorimotor and cognitive abilities. Indeed, some species of bats are more solitary^36^ whereas others reside in colonies exceeding tens of millions of individuals^37^, which could in turn influence the underlying neural computations. The emergence of methodologies that can provide accessibility to functionally^38^ and genetically^39^ diverse cell types in a wide range of species can help delineate their contribution to the different forms of behaviours bats exhibit. Finally, our approach can be extended to other social species exhibiting their own forms of collective behaviour. Competitive, cooperative or coordinated behaviours in groups of rodents^40,41^, primates^31^, schools of fish^42^, raiding ants^43^ and flocking birds^44^ are just some of the examples in which the ecology of groups necessitates precise monitoring and spatial localization of specific individuals^45^. Diversifying research across species using comparative studies^46,47^ can help to uncover the underlying neural mechanisms of collective behaviours.

## Methods

### Bats

Experiments involved a total of 20 adult male Egyptian fruit bats (*Rousettus aegyptiacus*; weight, around 150–190 g), in eight of which neural data were collected. The bats were distributed as follows: behaviour, five bats (social group 1), five bats (social group 2), two bats (object experiments); and neural recordings, five bats were implanted with a four-tetrode microdrive (three took part in foraging with social groups 1 and 2; two involved in the object experiment) and three additional bats were implanted with a miniaturized microscope (foraging with social groups 1 and 2). Experiments comprised 10–20 daily foraging sessions involving a group of 5–7 bats (social groups plus one or two implanted animals; the composition of the main electrophysiology datasets is shown in Extended Data Fig. 2a) or a group of three bats and a moving object. All of the animals were housed in humidity- and temperature-controlled rooms. Before the start of the experiments and between experimental epochs, the non-implanted animals were housed in a communal laboratory male colony. During the experimental period, the animals were housed in a separate housing room together with other bats. Non-implanted animals were housed in large cages, one for each social group. Implanted animals were initially single housed and subsequently, after recovery from surgery, co-housed in the large cages with the other bats. The lights in the housing room were maintained on a 12 h–12 h reverse light cycle (lights off–lights on, 07:00–19:00). All experiments were performed at the same time of day during their awake hours (dark cycle). All experimental procedures were approved by the Institutional Animal Care and Use Committee of the University of California, Berkeley.

### Collective behavioural set-up

All of the experiments were performed in an acoustically, electrically and radio-frequency-shielded room (5.6 m × 5.2 m × 2.5 m) with high-precision lighting control^48^, under uniform illumination (luminance level 5 lux), allowing animals to use both proximal (touch, olfaction) as well as distal (vision, audition) sensory cues. To minimize acoustic reverberation and dampen noise from the outside, the flight room ceiling and walls were covered with acoustic foam. An additional layer of acoustically absorbing black felt was used to protect the acoustic foam from being damaged by the bats while maintaining the intended acoustic environment. The flight room floor was also covered with the same acoustically absorbing black felt. The 3D spatial position of all of the animals was recorded using a modified version of a commercial RTLS (Ciholas). The system was composed of mobile tags (DWTAG100) that were identified and localized at a 100 Hz sampling rate by 16 static anchors (DWETH101), providing reference locations for the system (the arrangement of the anchors is shown in Extended Data Fig. 1b). Anchors and tags communicated through ultra-wideband pulses. One additional anchor (custom DWETH101) was used to record an external synchronization signal (see below). Tags were made of a lightweight (~2.9 g) transceiver and a LiPo battery, mounted onto custom made collars (~15 g total). A 16-bit three-axis accelerometer was included in the tag and could provide acceleration data at 100 Hz. The system communicated with a computer located outside the room through User Datagram Protocol (UDP) and was configured and operated through a web-based user interface running on Ubuntu v.18.04 Bionic. Data were recorded and saved using custom written scripts in Python v.3.9. The spatial resolution of the system was measured on a subset of the experiments by simultaneously tracking one or two bats with the RTLS, together with a highly precise camera-based system (Motion Analysis^24,25,48^) and was in the range of 10–20 cm (Extended Data Fig. 1c,d). For both electrophysiology and imaging, collective spatial experiments consisted of one of two types, each one permanently associated with a separate baseline group of five bats (social group 1 or 2) and differing only by the food source (bowl or feeders, see below). Implanted bats were added to the baseline groups after recovery from surgery. All of the bats were mildly food-restricted (>85% of their baseline weight) before the group sessions and often actively participated in the foraging experiment even when at their full weight, suggesting that food was not the main driver behind the active participation. In the case of bowl foraging (social group 1), a bowl or a plate of banana pieces was located close to the centre of the room, around 0.5 m from the ground, and the bats could spontaneously collect banana pieces from it. The bowl was typically filled with 60–100 g of banana and was occasionally replenished in the middle of a session. In the case of feeder-foraging (social group 2), four automated feeders placed on the wall at one end of the room dispensed a pureed fruit reward, as described previously^24,25,48^. A reward was triggered when a bat landed on the feeding platform and interrupted an infrared beam break sensor mounted in front on the reward port. Feeders were all independently controlled by an Arduino (Uno Rev3) and Adafruit Motorshield (1438; Adafruit) interfaced with a computer outside the experimental room. To encourage the bats to leave the feeders after the collection of food, we disabled a feeder after a bat triggered it. The next feed could be triggered by a different bat landing on the same feeder or by the same bat, after leaving the feeder and coming back (crossing a virtual barrier located 0.7 m from the feeders). We also carried out experiments involving a moving object that were similar to those described above with two main differences: (1) they involved three bats foraging from one single feeder (two of which were implanted with a tetrode microdrive); (2) they involved an object (a Styrofoam ball, 20 cm diameter; Extended Data Fig. 8a) that could be moved from outside the room using a cableway system and a pulley. The object was moved between two locations that the bats most often occupied: one close to a resting site (perch) and the other close to the feeder. To create a dynamic context, the ball was moved every 10–15 min between these two locations and some false starts and sudden movements of it were occasionally presented. Bats sporadically landed on the ball and often touched it around landing with the tip of their wings. The position and identity of each bat (and also of the object) were constantly monitored through the RTLS, interfacing with custom MATLAB scripts controlling the feeders. Foraging sessions lasted between 60 and 150 min and started with all of the non-implanted bats released from a small cage close to the entrance of the flight room. When implanted bats participated in the experiment, the group foraging session was flanked by two rest sessions, lasting 5–10 min, in which the implanted bats were kept—each one isolated—in a small cage (25 cm × 32 cm × 46 cm) within an opaque enclosure (40 cm × 46 cm × 65 cm) inside the flight room. Implanted bats were released from the small cage at the beginning of the foraging session, immediately after releasing the non-implanted bats. Neural activity was recorded during both group behaviour and rest sessions. Periodic clock pulses generated by a Master-9 device (A.M.P.I.) were used to create a timing signature that served as a common frame of reference for all of the recording systems (tracking, neural recordings and audio, see below).

### Microdrive implant procedure

Surgical procedures for electrophysiology implants were performed similarly to those described previously for Egyptian fruit bats^23,24,49^. A lightweight four-tetrode microdrive (Harlan 4 drive; Neuralynx) was implanted over the right hemisphere of each bat. Tetrodes were made of four strands of platinum-iridium wire (17.8 µm diameter, HML-insulated) and assembled as described previously^24^. Each of the four tetrodes was loaded into a telescoped assembly of polyamide tubes mounted into the microdrive and was individually moveable (~5 mm travel). On the day before surgery, the tip of each tetrode was cut flat and plated with Gold Plating Solution (Neuralynx) to reduce the impedance of individual wires to 0.2–0.5 MΩ. On the day of the surgery, anaesthesia was induced using an injectable cocktail of ketamine, dexmedetomidine and midazolam. The bat was then placed in a stereotaxic apparatus (Model 942; Kopf) and anaesthesia was maintained throughout surgery by injections (around once per hour) of an anaesthesia maintenance cocktail of dexmedetomidine, midazolam and fentanyl. The depth of anaesthesia was continuously monitored by testing toe pinch reflexes and measuring the bat’s breathing rate. The bat’s body temperature was measured using a rectal temperature probe and kept at approximately 35 °C through a regulated heating pad. After verification of effective anaesthesia, the skull was exposed, cleaned and the surrounding skin and tissue were retracted. During surgery, before placing the microdrive, the skull was scored to improve adhesion and mechanical stability. A bone screw (19010-00; FST), with a short piece of stainless-steel wire (0.008 inch, PFA-coated; A-M Systems) soldered to the screw head, was inserted into the skull in the frontal plate, and served as ground. Four additional bone screws (M1.59 × 2 mm, stainless steel) were placed into the skull for mechanical stability of the implant. A circular craniotomy of 2 mm was made in the skull above the hippocampus over the right hemisphere at 7 mm anterior to the transverse sinus that runs between the posterior part of the cortex and the cerebellum and 3.2 mm lateral to the midline. The craniotomy was covered with a biocompatible elastomer (Kwik-Sil; World Precision Instruments) until the microdrive was implanted. The skull and the base of the screws were covered with a thin layer of bone cement (C&B Metabond). Next, after removing the Kwik-Sil from the craniotomy and performing a durotomy, the microdrive was slowly lowered, with fully retracted tetrodes, to create a tight seal and the remaining exposed brain was covered with Kwik-Sil. Dental acrylic was applied to secure the microdrive to the screws and the skull. A ground wire from the microdrive was connected to the wire from the ground screw and covered with dental acrylic as well. All four tetrodes were initially positioned at approximately 800 µm below the cortical surface at the end of the surgery. Finally, the analgesic meloxicam (Metacam; Boehringer Ingelheim) was administered to the bat after surgery. Analgesics (3 days) and antibiotics (7 days) were administered daily after surgery, until complete recovery.

### Electrophysiology data acquisition, preprocessing and spike sorting

After surgery, tetrodes were lowered in small daily increments over a period of 1–2 weeks towards the pyramidal layer of the dorsal hippocampus (CA1 and CA2). The pyramidal cell layer was tentatively determined by the presence of high frequency ripples in the local field potential, concomitant with a transient (50–100 ms) increase in multiunit activity. All adjustments of the tetrodes were done while the bat was swaddled in a small fabric bag: neural activity from the tetrodes was monitored daily by connecting the bats’ microdrive to a wired recording system (Digital Lynx; Neuralynx) before the beginning of the experiments and after their completion. At the end of each recording session, one or more tetrodes were typically moved (20–160 µm) to sample—on the next day—from a different group of neurons, while ensuring maximal time for stabilization of the tissue. Tetrode positions were later verified histologically (see below). To record neural activity in freely flying bats, we used a wireless neural data-logging system (neural-logger; MouseLog16, vertical version, Deuteron Technologies). The logger was housed in a custom-designed 3D-printed case, together with the RTLS tag and two LiPo batteries (one for the logger and one for the RTLS tag; minimal duration, 150 min) and connected to the electrical interface board of the microdrive at the beginning of each recording. The whole system weighed around 15–17 g. Implanted bats used in our experiment weighed more than 150 g and could fly normally while equipped with the neural-loggers, as expected from previous experiments using wireless recording systems^9^. Electrical signals from the four tetrodes (16 channels) were amplified (200×), bandpass filtered (1–7,000 Hz), sampled continuously at a frequency of 29.34 kHz and stored on a SD card memory on the logger, with a voltage resolution of 3.3 µV. Wireless communication between the neural-logger and a static transceiver ensured proper synchronization and allowed basic monitoring and configuration through software (Deuteron Technologies). At the end of the recording session, data were extracted from the logger and saved. Spike sorting was performed as described previously^9,24^. In brief, recorded voltage traces were filtered (600–6,000 Hz) and putative spikes were detected by thresholding (3 s.d.) the filtered trace. Putative spike waveforms (32 samples, peak at the eighth sample) were used as input for the cluster sorting software (SpikeSort 3D, Neuralynx). Spike amplitude and energy were used as features for manual sorting. Unstable units, with visible drift in spike amplitude, and units coming from tetrodes that did not exhibit ripples in the local field potential, after careful evaluation of the whole session voltage traces, were discarded from the analysis. Only for the comparison between CA1 and CA2 (ref. ^50^) (Extended Data Table 1), units were classified as putative principal cells and putative interneurons based on spike width and average firing frequency, using similar criteria adopted from the hippocampus of rodents and bats^34,51^ (putative interneurons: average spike width < 0.4 ms or average firing frequency > 5 Hz, 11% of the recorded cells). Consistent with similar recordings from the hippocampus of rodents and bats, putative principal cells typically corresponded to elongated clusters in feature space and bimodal inter-spike-interval distributions, whereas putative interneurons corresponded to more symmetric clusters and unimodal inter-spike-interval distributions. A total of 373 well-isolated single units were recorded from the dorsal hippocampus of five bats (177 from experiments involving social group 1, and 77 from experiments involving social group 2 and 119 from the object experiment).

### Microscope description and implant procedure

The microscope used for wireless calcium imaging was similar to those described previously for Egyptian fruit bats^25^. In brief, the microscope is made of 3D-printed material (black resin; Formlabs) combined with commercially available optical and electrical components^52^ and assembled in our laboratory. Design files, part numbers and software are publicly available at GitHub (https://github.com/gardner-lab/FinchScope and https://github.com/gardner-lab/video-capture). Excitation light is emitted by a blue LED (470 nm peak; LUXEON Rebel) and collimated by a drum lens (45-549, Edmund Optics), before passing through the excitation filter (3.5 mm × 4 mm × 1 mm, ET470/40x; Chroma) and a dichroic mirror (4 mm × 6 mm × 1 mm, T495lpxr, Chroma). A gradient refractive index (GRIN) objective lens (GT-IFRL-200-inf-50-NC, GRINTECH) focuses the excitation light on the sample (0.5 NA). Fluorescence collected by the objective is transmitted through the dichroic mirror, an emission filter (4 mm × 4 mm × 1 mm, ET525/50m, Chroma) and focused by an achromatic doublet lens (45-206, Edmund Optics) onto an analogue CMOS sensor (MB001; 3rd Eye CCTV), acquiring at 30 Hz frame rate and 640 × 480 pixels. Frames can be streamed through a wireless transmitter–receiver couple (TX24019, 100 mW) and the entire system (LED, CMOS and transmitter) is powered by a lightweight consumer-grade 3.7 V, 300 mAh lithium polymer battery, which provided stable recording for about an hour at average imaging LED intensities (less than 100 µW post-objective power). The system is compatible with simultaneous streaming from multiple microscopes by using different carrier frequencies. The NTSC video and a synchronization signal (generated by the Master9, see above) were both digitized through a USB frame grabber and acquired using custom software^52^. The USB frame grabber was enclosed within a custom-made data acquisition box (DAQ) that could be connected to the receiver or directly to the microscope through a cable.

Surgical procedures were performed similarly to those described previously for Egyptian fruit bats^25^ and involved injection and implant surgery. Expression of the Ca-indicator GCaMP6f was mediated by pAAV9.hSyn.GCaMP6f.WPRE.SV40 (Addgene), injected into the dorsal hippocampus. In brief, following the same procedures for anaesthesia, analgesia and skull preparation described in the ‘Microdrive implant procedure’ section above, 1.25 μl of virus was injected at a rate of 4 nl s^−1^ above the desired coordinates (5.8, 2.8 and 2.6 mm in one bat (CA1) or 6.8, 3.2 and 2.8 mm in two bats (CA1–CA2), anterior to the transverse sinus, lateral to the midline and depth). The injection opening in the skull was filled with Kwik-Sil and the tissue was closed with sutures. Then, 4 weeks after the injection, implant surgery of a 1.8 mm diameter GRIN relay lens (130-004836, Inscopix) was performed according to the procedure described previously for Egyptian fruit bats^25^. The cortex above the dorsal hippocampus was aspirated using a vacuum pump attached to a 30 GA blunt needle. Sterile lactated ringer solution along with pressure from an absorbable sponge (Gelfoam, Pfizer) was applied to the brain to prevent bleeding during the aspiration. Aspiration continued slowly until the parallel fibres of the hippocampal oriens were visually identified. Before surgery, the relay lens was glued to the microscope through small bridges of light-cured flowable composite (Flow-It ALC, Pentron). The lens + microscope system was then slowly lowered, while imaging from it, until clear evidence of fluorescence from the target hippocampal region was observed, typically at about 100–200 µm from the tip of the lens to the dorsal surface of the hippocampus. Kwik-Sil was applied to seal the space between the lens and the edges of the craniotomy and carbon powder mixed with dental acrylic was applied around the surface of the skull and above the bone screws to hold the implanted lens in place. The glue bridges were carefully broken to separate the relay lens from the microscope, and the exposed surface of the relay lens was covered with Kwik-Sil while the bat recovered. Next, 2–3 weeks after lens implantation, the miniaturized microscope was aligned with the relay lens under anaesthesia as described previously^25^ and cemented in place. A custom 3D-printed protective housing case ensured protection from damage.

### Imaging data acquisition and ROI extraction

Wireless imaging videos were acquired through a custom made DAQ connected to a wireless receiver, communicating with a transmitter on the bat’s microscope. The transmitter, battery and tracking tag (see above) were all enclosed in a custom 3D-printed flight case. For each imaged bat, two DAQs simultaneously acquired the streamed frames to minimize streaming artifacts that depend on the relative position between transmitter and receiver. Artifacts typically impacted a small fraction of the frames of one receiver (mean, 1.43%) and rarely affected both receivers, provided that they were located in different positions. We recovered most of the artefactual frames by substituting them with their intact counterpart from the alternative receiver (see below). Raw videos (640 × 480 pixels) were acquired at 30 Hz and then spatially downsampled by a factor of two and temporally downsampled at 10 Hz. Preprocessing was performed using custom scripts in ImageJ (v.1.53c)^53^ and involved artifact detection and recovery, background compensation, motion correction, median filtering and spatial downsampling. In brief, one of the two acquired videos was selected as main (always from the same default DAQ) and the other as a backup; streaming artifacts from the main video were detected by a threshold criterion and impacted frames were substituted with the corresponding frames from the backup video. Next, large-scale background fluctuations were compensated by subtracting to each frame its gaussian filtered version (*σ* = 80 pixels) and rigid motion correction was performed (MOCO^54^). Temporal median filtering was applied (3 frames) and residual artifacts (mean, 0.13%), typically happening during rest, were treated as dropped frames and their fluorescence was interpolated. Finally, the videos were spatially downsampled by a factor of two, corresponding to about 2 µm per pixel. ROI segmentation and extraction of the fluorescence traces were performed similarly to previously published approaches^25,55^ and are therefore described in brief below. ROIs (putative neurons) were detected using an adaptation of a constrained non-negative matrix factorization approach designed for single-photon calcium imaging data (CNMF-E)^55^ and implemented in MATLAB. The following parameters were used for all extracted FOVs (gSig = 3, gSiz = 13, min_corr = 0.9, min_PNR = 50, ring_radius = 10, background_to_neuron_factor = 1.5, no spatial or temporal downsampling). Fluorescence traces were deconvolved using an autoregressive model (OASIS) with order *p* = 1 and using the ‘constrained foopsi’ method. Finally, identified ROIs were manually inspected to remove duplicates, inappropriate merges and non-cell-like ROIs. All of the subsequent analyses were performed on the inferred spike rate traces, normalized between 0 and 1 and smoothed with a 1 s moving average (normalized rate). Δ*F*/*F*_0_ traces shown in Fig. 4c were obtained by multiplying each raw temporal trace (C_raw) by a scaling factor proportional to the inverse sum of each ROI’s spatial footprint. Intensity correlation images (Fig. 4b and Extended Data Fig. 14a) were generated as part of the CNMF-E pipeline and show the local pixel correlations, reflecting correlated fluorescence emission by cell bodies and uncorrelated background.

### Histology

At the end of the electrophysiology experiments, bats were given a lethal overdose of sodium pentobarbital and perfused transcardially with 200 ml PBS (0.025 M, pH 7.4) followed by 200 ml of fixative (3.7% formaldehyde in PBS). During perfusion, the microdrive was left in place. Then, after a few minutes, the tetrodes were carefully retracted, the microdrive was removed and the brain was dissected and stored in the fixative solution for 1–2 days. The fixed brain was subsequently moved to a 30% sucrose solution in PBS overnight for cryoprotection. Coronal sections (thickness, 40 μm) were cut using a microtome (HM450, Thermo Fisher Scientific) with a freezing stage. Slices around the dorsal hippocampus and including the implant were stained for DAPI, the CA2-enriched protein PCP4 (for rodents see refs. ^56–61^) and the microglial marker IBA1 (to highlight tetrode tracks). In brief, slices were permeabilized in PBS + 0.3% Triton X-100 (PBS-X), followed by incubation in blocking solution (PBS-X + 10% donkey serum). After overnight incubation at 4 °C with primary antibodies (goat anti-IBA1, 1:500, ab5076, Abcam; and rabbit anti-PCP4, 1:200, HPA005792, Sigma-Aldrich), the slices were washed in PBS-X and incubated for 120 min at room temperature with secondary antibodies (donkey anti-goat Alexa-647, 1:1,000, Invitrogen, A32849; donkey anti-rabbit Alexa-488, 1:1,000, Invitrogen A-21206). DAPI (1:10,000, Thermo Fisher Scientific) was added for the last 10 min of secondary incubation. The sections were washed in PBS-X and cover-slipped with aqueous mounting medium (ProLong Gold Antifade Mountant, Thermo Fisher Scientific). Fluorescence images of each section surrounding the implant were acquired using the Axioscan Slide Scanner (Zeiss) and used to localize tetrode tracks relative to hippocampal subfields (CA1 and CA2). Putative CA2 was identified on the basis of a combination of PCP4 fluorescence, DAPI staining and correspondence with the brain atlas of this species of bats^62^. Tetrode positions were determined by serial reconstruction of the tetrode arrangement in adjacent coronal sections. As tetrode tracks were not perfectly parallel to the coronal cutting plane, the path of each electrode could be visualized (IBA1 staining) as elongated segments of inflamed tissue in each section. The tip of each electrode was found by tracking the tissue gliosis across anatomically arranged coronal sections. Ten out of a total of twelve tetrodes (across three microdrives) were successfully identified and localized in the dorsal hippocampus of implanted bats. The remaining two tetrodes provided putative hippocampal units (and visible hippocampal ripples) and were included in the analysis but could not be associated with a specific location (comprising a total of 7 out of 254 units). Similar procedures were applied to confirm the location of the tetrodes in the hippocampus for the bats recorded in the object experiment. Similar procedures—with the exclusion of staining for PCP4 and IBA1—were performed at the end of the imaging experiments to confirm the lens targeting accuracy and the GCaMP6f expression profile around dorsal hippocampal regions CA1 (one bat) or CA1–CA2 (two bats).

### Recording and detection of echolocation calls

Sounds in the experimental room were recorded using a dedicated ultrasonic microphone (Earthworks M50, Earthworks) mounted on one side on the room, which was connected to a preamplifier (OctaMic II, RME Synthax) and recorded audio data at a sampling rate of 192 kHz. The microphone output was corrected to achieve flat-frequency responses up to 96 kHz. Audio recordings were controlled using the Soundmexpro (HorTech) toolbox for MATLAB (MathWorks) and recorded using custom MATLAB scripts. Detection of echolocation calls was similar to ref. ^48^ and was performed as follows. Downsampled audio data (96 kHz) were band-pass filtered (10–40 kHz) and *z*-scored. All events larger than 10 s.d. were considered to be potential echolocation clicks and identified with the MATLAB function findpeaks, with a minimum peak distance of 10 ms. Other wide-band signals could contaminate the detection of clicks but were much rarer than the thousands of echolocation calls typically emitted in one session. To account for this, we took advantage of the similarity of echolocation clicks in the spectral domain (for this species of bats^32^) and looked for the most numerous cluster (*k*-means, 4 clusters) in the space defined by the first 3 principal components of the power spectrum of all putative clicks. The correspondence between this cluster and actual echolocation clicks was confirmed by the presence of two prominent peaks in the inter-click-interval distribution, consistent with what is expected for this species^32^ (intra-pair interval, ~20 ms; and inter-pair interval, ~100 ms; Extended Data Fig. 12a). The detected echolocation signals were largely consistent with their production by the flying bat as (1) their rate increased in amplitude when a bat approached the microphone (Extended Data Fig. 12d), (2) there was an increase in echolocation jamming with increasing numbers of flying animals (Extended Data Fig. 12e) and (3) there was a tight temporal alignment between detected echolocation clicks and the wing-beat cycle of the flying bat^9,63^ (Extended Data Fig. 12f).

### Data analysis

All analyses were conducted using custom code in MATLAB (2021a, MathWorks).

### Processing of positional data during group behaviour

#### Preprocessing of tracking data and basic analysis of positional features

The positions of all bats recorded by the RTLS were smoothed using local quadratic regression (1 s window). The tracking quality was further improved by considering that, when not flying, our bats did not typically change their position by crawling, therefore remaining in the location where they previously landed. We therefore detected flight epochs based on the prominent 8 Hz component in the accelerometer signal (wingbeats) and used them for excluding all flights from a second smoothing step: all tracking data during rest (that is, not associated to wingbeats) were further smoothed with a moving median (5 s window). For each bat, flights were identified on the basis of a velocity threshold of 0.5 ms^−1^ and used to segment a bat’s session into rest and flight epochs. Bats tended to rest in a handful of positions, typically—but not exclusively—around the upper corners of the room (Fig. 1b). We therefore clustered positions during rest for each bat using agglomerative hierarchical clustering with a minimum of 10 s occupancy and 0.2 m linkage distance (Extended Data Fig. 2d). Occupancy maps were calculated by counting the number of samples spent by a bat in each 2D spatial bin (21 × 21 bins, ~0.3 × 0.3 m). No smoothing was applied. Exploration ratio during rest (Extended Data Fig. 2c) was calculated as the fraction of bins visited by each bat (only contour bins were considered, minimum occupancy 5 s). The fraction of time close to the feeder was calculated as the time in the session spent at a distance of <0.3 m from the feeder locations (Extended Data Fig. 2e). Correlations between spatial preferences (Extended Data Fig. 2f) were calculated as the Pearson correlation between rest occupancy maps (unrolled in 1D) across subsequent sessions or between different bats. Heading was defined for each flight sample as the direction of the instantaneous velocity vector in the *xy* plane.

#### State-space analysis

A configuration of *N* bats in a given sample was defined as the vector of all positions (*r*_1_, *r*_2_, …, *r*_*N*_) and served as an input for state-space embedding by dimensionality reduction. Epochs in which all bats were resting were extracted and downsampled to one configuration every 3 s. This interval was chosen because it was approximately equivalent to the time between two flights (that is, a change in the group configuration). Euclidean distances between pairs of configurations were calculated and used as inputs for Sammon projection in two dimensions^64^, thereby obtaining a point in 2D (state space) for every input configuration of the group. Occupancy in the state space (Fig. 1c) was calculated on 80 × 80 spatial bins covering the range of obtained states and smoothed with a Gaussian kernel (*σ* = 1 bin). Considering the sparsity in the state space (that is, the highly clustered spatial preferences of the bats), states could also be approximated as discrete variables as follows. We pooled together all of the positions occupied by the bats during epochs of general rest and clustered them using agglomerative hierarchical clustering with a minimum of 10 s occupancy and 0.2 m linkage distance, therefore defining a set of discrete observed locations. In this way, each sample of general rest was associated with a configuration of discrete values (corresponding to the combination of bat identity × positional cluster identity). All of the possible states were calculated as (number of positional clusters)^(number of bats)^. All visited states were defined as the effectively observed combinations, whereas all frequent states were defined as the configurations with occupancy higher than the s.d. of all of the state occupancies for that session (Fig. 1d, dashed line).

#### Social network and proximity indexes

The spatial proximity between pairs of bats in a given session was quantified as the fraction of time in which the inter-bat distance was lower than 0.3 m. This value was corrected by considering that bats could be found in close proximity as a consequence of shared spatial (rather than social) preferences. Thus, we also calculated the average chance proximity for the same pair, by randomly circularly shifting in time the position of one of the bats in the couple. The chance distribution was generated by repeating this procedure 1,000 times. We calculated two measures of social proximity by comparing the empirical value of the spatial proximity and its chance distribution: a proximity index (corresponding to the difference between the empirical value and the mean of the chance distribution; Fig. 1g) and its associated *P* value (the fraction of shuffled spatial proximities that exceeded the empirical value; Fig. 1f).

### Place-fields and spatial information

#### Spatial information in 2D

For the analysis of spatial firing fields across all flights, we considered only active cells (*n* = 147 from three bats), with a minimum firing rate of 0.2 Hz during flight (minimum of 5 flights, at least 3 flights with spikes) and a minimum exploration ratio of 0.5 (as defined above, but across the whole room surface, see the ‘Preprocessing of tracking data’ section). We focused on the spatial firing in the *xy* plane (parallel to the ground), where most of the positional variance was concentrated. To compute 2D place-cell firing-rate maps, we projected all positions during flight onto the *xy* plane and calculated the occupancy-normalized firing rates as follows: we binned the 2D area of the room into fixed-sized spatial bins (0.15 × 0.15 m^2^) and calculated the occupancy (time spent in each bin) and the number of spikes fired in each bin. We smoothed both the spike-count map and occupancy map with a Gaussian kernel (*σ* = 1.5 bins) and calculated their ratio, bin by bin, therefore obtaining the firing rate per bin. Spatial bins in which the bat spent <200 ms were invalidated (white in Fig. 1j and Extended Data Fig. 4a), unless surrounded by at least one valid bin. Spatial information per spike^65,66^ was calculated by summing across all valid bins:

$${\rm{SI}}=\sum _{i}\frac{{p}_{i}{\lambda }_{i}}{\lambda }{\log }_{2}\frac{{\lambda }_{i}}{\lambda },$$

where *p*_*i*_ is the probability of being in bin *i*, *λ*_*i*_ is the firing rate on the same bin and *λ* = *Σ*_*i*_*p*_*i*_*λ*_*i*_ is the average firing rate across all bins. A shuffling procedure was used to classify a cell as significantly spatially informative by comparing the empirical value of the spatial information to a spike-shuffled distribution. The shuffled distribution was generated by randomly shifting the timestamps of the cell’s spike-train circularly (after cutting rest epochs) and was repeated 1,000 times for each neuron. Significant place cells were defined as active neurons for which the empirical value of the spatial information exceeded the upper 95% confidence interval of its shuffled distribution.

#### Spatial information in 1D (flight paths)

As previously observed for solo bats or pairs of bats^24,25^, many flights of our animals followed along similar paths, typically traversed in only one direction. We took advantage of this feature and calculated spatial firing maps along tightly confined repeated trajectories (referred to as 1D flight paths). Flights were clustered into similar paths by using an analogous approach to that described previously^25^. In brief, flight trajectories were spatially downsampled to seven points per flight (first and last points corresponded to the take-off and landing positions, respectively). The Frechet distance^67^ between downsampled flights was used as a measure of flight similarity and similar flights were clustered by agglomerative hierarchical clustering. The linkage distance was set to 1.1 m after manual inspection of flight groupings. Spatial firing fields along flight paths (1D fields) were calculated for each repeated path and neuron with at least five flights and a minimum of four flights with spikes (*n* = 132 cells from three bats for collective foraging experiments and *n* = 116 cells from two bats during the object experiment). To compute the 1D fields, we used a similar procedure to the one adopted for 2D maps, applied in this case in only one dimension, to flight paths as 1D parametric trajectories rescaled between take-off and landing (bin size = 0.15 m). The firing rate was smoothed with a Gaussian window (7 samples) and spatial information was calculated across 1D bins as described above. A shuffling procedure was used to assess the significance of the spatial information of each 1D field. Similarly to what was described for 2D maps, spatial information was calculated on a shuffled spike distribution, generated by randomly shifting the timestamps of the cell’s spike train circularly (considering only flight epochs from the analysed path). Shuffling was repeated 1,000 times for each neuron and path (1D field). Significant 1D fields were defined as those for which the empirical value of the spatial information exceeded the upper 95% confidence interval of its shuffled distribution after applying Bonferroni correction for the number of paths examined for that neuron. The stability of 1D fields within a session (Fig. 1l) was measured by splitting each path into two random halves of repeated flights, separately calculating 1D fields on each half (Extended Data Fig. 4b) and calculating the Spearman correlation between corresponding halves.

### Social modulation of firing during flight of the recorded bat

Social modulation of firing during flight of the recorded bat was tested using three complementary approaches: (1) a stepwise GLM; (2) a test for firing differences between social and non-social flights anchored to specific take-off and landing locations—explicitly controlling for positional changes; and (3) a conservative test using modulation scores that aimed to identify social modulation in cases of minimal changes of kinematic variables (position, head direction and acceleration). We focused on the time periods around take-off and landing because these were associated with the largest fraction of spatial selectivity, and, furthermore, take-off and landing locations naturally constituted points in space were behavioural and positional variability was minimal (Fig. 1b), therefore enabling rigorous assessment of social modulation. Flights were divided into social and non-social using an empirically derived distance threshold (Fig. 2a), unless otherwise stated: all flights landing closer to a bat than 0.6 m were classified as social, whereas all flights landing further than 0.9 m were classified as non-social. The three approaches are explained below and are consistent with a significant modulation of hippocampal activity by the social nature of the bat’s spatial behaviour.

#### Social modulation for social versus non-social flights

*Approach 1 using GLM*. The aim of this analysis was to test whether the social nature of a flight had significant explanatory power in predicting the firing rate of hippocampal neurons across all of the flights executed by the recorded bat in a given session, under the null hypothesis that simpler models, including the position of the recorded bat as explanatory variable, could fully explain the variance in firing rate. Social modulation of firing was tested across all flights using a stepwise GLM^68^. The stepwise procedure aimed to build a simple model that tries to explain firing rate around take-off or landing—for all of the flights—with the fewest possible explanatory variables. We allowed for the fact that firing modulation induced by the social nature of a flight could happen, for each cell, at different timepoints around take-off and landing, and first determined the optimal time bin for each neuron. We therefore first divided flights into social and non-social on the basis of the nearest-neighbour bat distance at landing (see above). We next systematically tested for differences in the mean firing rate between social and non-social flights using a sliding window of 500 ms around take-off and landing ([−1 s, +1 s], 100 ms increments) using the conditional *C*-test for Poisson means^69^. For each cell, we then considered the time bin with the lowest *P* value for further analysis. We built a model using the number of spikes in the optimal time bin as a response variable and three explanatory variables: *x* and *y* position of the recorded bat at the centre of the optimal time bin (positional coding) and the social nature of the flight (social versus non-social, using the distance for non-social flights to >0.6 m to include all of the flights in this case). We considered only active cells (*n* = 162 from three bats), with a minimum average firing rate of 0.2 Hz throughout the session, and included only sessions with a minimum of 40 flights (total) and 20 flights per category (social, non-social). We used the MATLAB function stepwiseglm, considering a Poisson distribution as the distribution of the response variable and the log function as the link function. The stepwise procedure starts from a constant intercept model and iteratively adds or removes terms (*x*, y and social category) on the basis of their statistical significance in explaining the response variable. The comparison is performed on incrementally larger models using a deviance test^68,70^, which tests whether a model including more explanatory variables performs significantly better than a simpler model. This enabled us to compute the percentage of cells in which the social nature of the flight yielded a significantly better model than simpler models including only position and/or a constant term.

#### Social modulation for social versus non-social flights

*Approach 2 based on firing differences with no positional differences*. The aim of this analysis was to test whether the social nature of a flight could cause a significant change in firing rate around specific take-off and landing locations (in contrast to the previous analysis that considered all take-off and landing spots), while controlling for changes in the position of the recorded bat at the time of the modulation. We took advantage of the fact that take-off and landing locations were naturally clustered in space and systematically evaluated the activity of neurons around these anchoring points, iteratively applying the same procedure to the pairs defined by a neuron and an anchoring location. First, we applied spatial clustering to all of the resting locations of the recorded bats (see above) and retained only flights from clustered locations (corresponding to most of the flights; Extended Data Fig. 2d). As a result, we obtained a series of positional clusters at take-off and landing with each corresponding to repeated flights diverging from or converging on a restricted set of positions. Then, for each positional cluster, we applied the following procedure. We classified social versus non-social flights as described above and retained only positional clusters with a minimum of five flights per category. We next systematically tested for differences in mean firing rate between social and non-social flights on a sliding window of 500 ms around take-off or landing ([−1 s, +1 s], 100 ms increments), using the conditional *C*-test and applying Bonferroni correction for the number of tested windows. For each cell and in the case of take-off locations, we considered the time bin with the lowest *P* value as the optimal time bin for further analysis. In the case of landing, we instead prioritized time windows entirely confined before landing and considered windows after landing only if no significant firing difference was found before. We included only cells with a minimal average firing rate of 1 Hz in the optimal time window across all flights (*n* = 135 from three bats). Once the optimal time bin was found, corresponding to a significant difference between the firing rate on social versus non-social flights, we tested for significant differences in the position of the recorded bat at the centre of the optimal time bin. This was done by separately testing for differences in the *x*, *y* and *z* during social versus non-social flights using a simple permutation test. We considered a significant difference in position if any of the three coordinates showed a *P* value smaller than 0.05. Percentages of cells are reported in the main text.

#### Social modulation for social versus non-social flights

*Approach 3 based on modulation scores*. The aim of this analysis was to find, following a more conservative approach, neurons that showed consistent modulation of firing rate during social versus non-social flights while at the same time exhibiting minimal changes in position, heading direction and acceleration. To do so, we implemented a procedure (modulation score calculation; Extended Data Fig. 6) that enabled us to (1) minimize any potential unbalance between the number of social versus non-social flights and (2) test differences between the four variables of interest (firing, position, heading and acceleration) within the same statistical framework. The calculation of the modulation scores was performed iteratively, following the same steps for all of the active neurons and all of the anchoring points at take-off and landing fulfilling the inclusion criteria (see below). We divided flights into social and non-social, as described above, and required a minimum of five flights for each category for further analysis (median of 12 versus 9 flights per category). First, we identified the optimal time bin in which we could find a significant modulation of firing based on the social nature of the flights, using the same procedure described above (again applying Bonferroni correction for the number of tested windows). We included only cells with a minimal average firing rate of 1 Hz in the optimal time window across all flights (*n* = 135 from three bats). Once the optimal time bin was found, we calculated the number of spikes within the time bin (500 ms), as well as the position and heading angle of the recorded bat at the centre of the time bin and the average absolute acceleration (provided by the onboard accelerometer) within the time bin for each flight. Next, we selected a subsample of all of the available flights, made of five randomly selected social flights and five randomly selected non-social flights and calculated (1) the difference between the average firing rates; (2) the distance between the average positions; (3) the difference between the average heading angles; and (4) the difference between the average accelerations, where averages were calculated for the same five social versus five non-social flights. Each of these four differences was considered as the empirical value for that particular subsample and compared with 100 shuffled sets obtained by randomly permuting the social nature of flights. A variable of interest (firing, position, heading or acceleration) was considered to be significantly different for that particular subsample if the corresponding difference between social and non-social flights was larger (in absolute value) than 95% of the corresponding shuffled values. We repeated this procedure 100 times, each time sampling a different subset of five social versus five non-social flights and each time calculating the significance of the differences in firing, position, heading and acceleration. The modulation scores for each examined cell and anchoring location were calculated as four numbers (firing, position, heading, acceleration) quantifying the faction of subsamplings in which the corresponding variable showed a significant difference (for example, a firing modulation score of 0.7 means that in 70 out of 100 subsamplings we found a significant difference in average firing between social and non-social flights for that particular cell around a given location). We considered a cell as modulated by the social nature of a flight—with minimal changes in position, heading and acceleration—if the firing modulation score was larger than 0.5 (average 0.7 across all modulated cells; Extended Data Fig. 7) and all of the remaining scores (position, heading and acceleration) were smaller than 0.5 (average 0.2 across all modulated cells; Extended Data Fig. 7). The modulation score for heading was not considered for time bins centred before take-off or after landing (minority), as heading was not defined on those bins. The modulation value (as opposed to the score) of the firing rate for a neuron around a given anchoring point (absolute value in Fig. 2d,g,k and signed value in Fig. 2l) was calculated as the average difference in firing between social and non-social flights across all subsamplings (corrected by the mean of the shuffled distribution, typically equal to zero), divided by the average firing frequency for that cell over the entire session and expressed in percentages. Although this approach using modulation scores provides an underestimation of the fraction of cells that is modulated by social factors (as it effectively excludes cells that are also modulates by other factors, such as acceleration and heading that can also be socially modulated), it nonetheless provided a complementary assessment for dissociating movement and positional modulation from social modulation.

#### Responses to conspecifics versus responses to a moving object

The analysis of experiments involving three bats and a moving object was performed according to similar procedures as described in the above sections and, importantly, using the exact same method (modulation score, approach 3). To avoid contamination from social responses, modulation by the object was evaluated considering exclusively flights landing on an empty spot or close to the object when no bats were present. For each cell and take-off or landing location, as described above, we followed an iterative procedure for finding the optimal time bin for further analysis and similar criteria on minimum number of flights and spikes per flight were adopted. In general, single-unit response profiles around the same location tended to be largely different when an object was present versus when a bat was present at landing (84% bat modulated and 71% object modulated showed significantly different responses, *P* < 0.05 empirical data versus shuffled flight identities; some example responses are shown in Extended Data Fig. 8d).

#### Social modulation around flights to a specific target bat

The aim of this analysis was to investigate whether the modulation of firing during social versus non-social flights could be explained by the presence or absence of a specific bat at the landing spot, regardless of other bats. To test this hypothesis, we simply repeated the above analysis by replacing the nearest-neighbour bat distance at landing with the distance to a specific bat (termed as the target bat), iteratively testing all of the bats that participated in the session (minimum of 5 flights to target bat: landing distance <0.6 m; minimum of five flights not to target bat: landing distance >0.9 m). We adopted this approach following the structure in the bat group behaviour where the positions of different bats in specific locations were often correlated. Yet this approach enabled us to identify the cases in which sufficient instances occurred for each specific target bat to assess potential identity selectivity (points with low ‘fraction same’ in Fig. 2l,m). Thus, the analysis below is mainly aimed at understanding whether the effects obtained under the assumption that a particular bat was the main driver of the modulation are comparable to or larger than the effects obtained under the assumption that any bat was driving the modulation. We followed the analyses described above for each target bat with enough flights (see above, *n* = 142 cells from three bats). First, we looked for cells with significant differences in firing and no significant differences in position (approach 2). Second, we followed the more conservative exclusion approach (modulation scores, approach 3; see above). As a result of the iterative nature of the procedure, a cell could be modulated by different target bats and around different anchoring locations (a triplet: {cell, location, bat}). However, we found that most of the cells were modulated around a single position (79%) and for a single target bat (75%; Extended Data Fig. 9i), underscoring the selective nature of the hippocampal neurons’ activity. In additional analyses that are shown in Fig. 2l,m, we assessed the significance of the change in firing rate modulation for a specific target bat compared with the general distinction between social versus non-social flights (Fig. 2l) and compared with other bats (Fig. 2m). For assessing the significance of change in firing rate modulation with to other bats, we computed for each neuron showing significant social modulation for a given target bat the firing modulation score that would correspond, in the same time bin, to a different target bat (we restricted the analysis to cells with a minimum eight flights to target, eight flights not to target; Fig. 2m) and tested whether the two were significantly different (Wilcoxon signed-rank test). We also calculated the percentage of flights that had the same class (to target, not to target), when classified based on the original target bat and when based on the different target bat, as two bats could potentially be always present in the same location together (Fig. 2l,m, colour code). In a similar analysis (Fig. 2l), we examined in which way the modulation value obtained for a specific bat was different from that obtained for social versus non-social flights in the same time bin. To test for significant differences between the two conditions, we excluded 6% (5 out of 88) of the pairs in which the firing modulation value changed sign (going from increase to suppression or vice versa when considering social, non-social instead of to target, not to target) and again tested for significant differences between the two values (Wilcoxon signed-rank test on the absolute values). For the analysis of social proximity between the recorded bat and different target bats (Fig. 2n), we calculated the proximity index (a purely behavioural measure, see above) between the recorded bat and the target bat (the one associated with significant modulation of hippocampal activity) and between the recorded bat and a ‘different bat’ (not associated with a significant change in firing rate in the same time bin and location), therefore obtaining two values for each cell. To avoid ambiguities, we included only cells modulated by a single target bat in one single location (*n* = 30 from three bats) and considered a unique different bat, fulfilling inclusion criteria for minimum number of flights and associated with the largest number of flights (meaning that the different bat could be tested, but did not cause significant modulation of firing). The difference between proximity indexes was tested by pairwise comparison (Wilcoxon signed-rank test). To show the magnitude of the effect, the proximity indexes in Fig. 2n were normalized to the median proximity index between the implanted bats and all other bats, regardless of neural modulation. For all socially modulated cells, we controlled for differences between the time in the session when flights to target versus flights not to target occurred (only 8 out of 58 cells showed a significant difference between the time of flights, *P* < 0.05, Wilcoxon rank-sum test), suggesting that, overall, the modulation was not due to unstable recording of neural activity throughout the session, consistent with the fact that firing patterns were generally very stable (Fig. 1l).

### Simulated spatial, social and conjunctive responses

Simulations were performed by combining the observed behaviour of implanted bats during collective foraging with modelled cell responses. All of the sessions that were included in the analysis of single-unit responses were also used for simulations. We performed two sets of simulations: one for evaluating the responses of different cell classes and one for evaluating the decoding performance from the activity of simulated cells. The two sets differed only in the number of simulated cells of each type and in the distribution in space and time of spatial and social responses (described below). The firing rate of each neuron was modelled on 200 ms time bins as an inhomogeneous Poisson process with rate *λ*(*t*) given by the contribution of spatial and social responses. In particular, for each time bin centred at *t*_*i*_:

$$\lambda \left({t}_{i}\right)={\lambda }_{{\rm{spont}}}+{\lambda }_{{\rm{spatial}}}({t}_{i})+{\lambda }_{{\rm{social}}}({t}_{i})$$

$${\lambda }_{{\rm{spatial}}}\left({t}_{i}\right)=w\left({t}_{i}\right){\lambda }_{c}{{\rm{e}}}^{\frac{{\left(x\left({t}_{i}\right)-{x}_{c}\right)}^{2}}{2{\sigma }_{c}^{2}}}$$

$${\lambda }_{{\rm{social}}}\left({t}_{i}\right)=b\left({t}_{i}\right){\lambda }_{s}{{\rm{e}}}^{\frac{{\left({t}_{i}-{t}_{s}\right)}^{2}}{2{\sigma }_{s}^{2}}}$$

The above equations describe spatial fields with maximal firing *λ*_*c*_ at the centre *x*_*c*_ of the 2D field and width *σ*_*c*_. *w* is a windowing function that ensures firing at rest decays within 0.5 s from take-off or landing. ‘Social’ responses were simulated as Gaussian-shaped changes in firing rate (value *λ*_*s*_) happening around take-off or landing (*t*_*s*_) and lasting for *σ*_*s*_ seconds, conditioned to the presence or absence of a bat at the landing spot (*b* term), using a distance threshold of 0.6 m. Different cells classes could be generated by modifying the parameters of the model. Canonical place cells were generated by setting *λ*_*s*_ = 0; pure social cells were those with *λ*_*c*_ = 0; conjunctive cells were defined by setting *λ*_*s*_ = ±*λ*_*c*_ and *b*(*t*_*i*_) true only if (*x*(*t*_*i*_) − *x*_*c*_)^2^ *≤* *σ*_*c*_^2^, that is, firing was modulated both positively or negatively by the presence or absence of a bat, but only within a given spatial field (the sign of the social modulation was positive with 0.5 probability). To evaluate the general properties of modelled cells (Extended Data Fig. 10e), we simulated 50 cells per session and per implanted bat. These cells were randomly selected from the three categories defined above with probabilities 0.2 (spatial), 0.2 (social) and 0.6 (conjunctive), given the sparser responses of conjunctive cells. Parameters of the model such as the baseline firing rate, field width, firing rate at the centre of the spatial field or rate change for social versus non-social flights were derived from the experimentally observed values (*λ*_spont_ = 0.4 Hz, *λ*_*c*_ = 8 Hz for spatial cells, *λ*_*s*_ = 4Hz for social cells, *λ*_*s*_ = ±*λ*_*c*_ = ±4 Hz for conjunctive cells, *σ*_*c*_ = 50 cm, *σ*_*s*_ = 0.5 s). *t*_*s*_ was randomly selected to be at take-off or landing, whereas *x*_*c*_ was uniformly distributed across the 2D extension of the room. Activity from the simulated cells was analysed with exactly the same methods adopted for real cells and used to quantify spatial tuning and social modulation. For the second set of simulations (Extended Data Fig. 10f), 100 cells were simulated for each session and implanted bat, chosen at random from the three categories (~33 cells per class per session and implanted bat). Given that we were interested in decoding the landing position and the social nature of flights at landing, *x*_*c*_ was randomly sampled from the relevant landing locations of the bats (min 5 flights) and *t*_*s*_ was fixed at landing only, such that spatial, social and conjunctive responses were all concentrated around landing. Decoding of the landing location and or of the social nature of flights was performed by training multiclass support vector machines, by using the average activity of simulated cells on a [−0.5, 0.5] s time window around landing. Decoding accuracy was evaluated by fourfold cross-validation for increasing the numbers of cells from each of the modelled classes.

### Analysis of alternative reward-related explanations for the social modulation

We carefully examined several alternative explanations for the social modulation involving the interplay between identity and reward (Extended Data Fig. 11). First, we looked for evidence of leading-following dynamics (Extended Data Fig. 11a), whereby flights of a given bat to reward locations reliably preceded or followed flights of another bat to the same location. We tested each pair (bat_*i*_, bat_*j*_) twice, corresponding to one bat leading and the other following or vice versa. We quantified the interval between bat_*i*_ landing on a reward location and bat_*j*_ taking off towards the same location, and we compared the median of observed intervals with that of a shuffled distribution in which the take-offs of bat_*j*_ were circularly permuted, therefore preserving the inter-flight time. We repeated this process 100 times and calculated the fraction of shuffled sets yielding a median interval smaller than the empirical value. To consider a relationship as significant, we required the aforementioned fraction to be <0.05 and a minimum of 5 intervals between landing and take-off shorter than 12 s for each session. Next, we tested different explanations for the modulation of neural activity potentially associated with reward and described in Extended Data Fig. 11b–e. Potential reward blocking was tested by looking at the landing locations at which cells were modulated (Extended Data Fig. 11b); potential disturbing (scrounging) was examined by looking at the take-off locations preceding landing at which cells were modulated (Extended Data Fig. 11c). In both cases, we found little overlap between the locations that would be expected if only the reward caused the response. Next, potential ‘next reward availability’ was tested by looking at the probability and timing of next flights to reward when the target bat was either present or absent at the location where cells were modulated (Extended Data Fig. 11d); potential disturbance of the target bat was investigated by looking at the distribution of the time intervals from last reward of the target bat relative to the time of neural modulation (Extended Data Fig. 11e). Finally, the percentage of cells directly responsive to the reward was calculated for a subset of the single units, recorded with a probabilistic reward delivery and with at least 5 rewarded and 5 unrewarded flights (mean 42 versus 29, respectively; 73 neurons from two bats). The average firing profile was calculated in a [0–3] s time window after landing for trials with or without reward delivery (happening ~1 s after landing), when the bat landed on the same reward location in the absence of any other bat. The absolute difference, across the window, in the average number of spikes between rewarded and unrewarded flights was compared with 100 shuffled values of the same difference obtained by a similar procedure, where the identities of rewarded and unrewarded flights were randomly permuted. A cell was considered to be significantly reward responsive if less than 5% of the shuffled values were larger than the empirical value.

### Social modulation of firing during others’ flights, when the recorded bat was stationary

Modulation of neural activity under stationary conditions was evaluated around take-off of other bats. To avoid ambiguous events, we considered an interval from −1 to +1 s around each take-off and excluded all take-offs associated (1) with intersected intervals or (2) with intervals in which the recorded bat was also flying, which were very rare. All of the remaining intervals were therefore associated to the take-off of one single bat and disjointed with other intervals (the distribution of the number of valid events per session is shown in Fig. 3a). To account for the fact that hippocampal activity could be influenced by the overall movement of the recorded animal, in our main analysis, we considered only events characterized by low mobility of the recorded bat in the [−1s, +1s] interval around take-off (all flights and periods of high mobility are shown in Extended Data Fig. 13). To assess the recorded bat’s mobility, we used an on-board accelerometer. We calculated the vectorial norm of the three-axis acceleration recorded from the accelerometer and subtracted it to *g* (9.81 m s^−2^). All flights in which the absolute deviation from *g* exceeded 0.03 m s^−^^2^ in any of the samples of the considered interval were excluded (Fig. 3b). Only cells with a minimum of 20 low mobility events (mean across cells: 155) and a minimum average rate of 0.2 Hz across the session were considered for further analysis (*n* = 177 from three bats). This enabled us to effectively behaviourally clamp the recorded bat and exclude modulation of neural activity by self-movement. Significant modulation was assessed by systematically comparing firing rates in a sliding window around others’ flights (500 ms duration, 100 ms steps, range: −1 s, +1 s around take-off), with the average firing rate across the whole interval. A cell was defined to be significantly modulated if the firing rate was significantly different from the average firing in any of the tested time windows (*P* < 0.05, Wilcoxon signed-rank test after Bonferroni correction for the number of tested windows). Comparison of hippocampal responses around others’ flights with or without echolocation (Extended Data Fig. 13e) was carried out for the subset of significantly modulated cells (as defined above) that contained enough trials of both types (minimum of 20). The delay between responses was calculated as the time lag giving the maximal cross-correlation between response profiles, considering only cells with maximal cross-correlation larger than 0.2 per sample. The click-triggered PSTH (Extended Data Fig. 13f) was calculated for the significantly modulated cells by considering the firing rate around echolocation clicks (minimum of 150 clicks, separated by at least 80 ms to avoid double counting within a click pair) emitted when no bats were flying and normalized to the baseline firing rate. For generating the shuffled trace, the time instants corresponding to the number of detected clicks were randomly sampled from the same epochs of no flight. The magnitude of the modulation for close versus far take-offs (Extended Data Fig. 13d) was measured for each cell as follows: first we selected the subset of cells with enough repetitions (minimum 10) of both low mobility close flights (<1 m take-off distance) and low mobility far flights (>1 m take-off distance). We then calculated the average firing rate profile around take-off events ([−1s, +1s]) for the two classes. The ‘magnitude’ of the modulation was then computed for each cell as the scalar product between the average firing rate profile (close versus far) and a template response, defined as the average firing rate of all modulated cells rescaled between −1 and 1. A similar definition, through a scalar product, was used to calculate the magnitude of the response for selectivity index calculation (see below) and for the distinction between CA1 and CA2 responses (Extended Data Table 1). The selectivity index of social modulation during others’ take-off was calculated for three different conditions: (1) selectivity for the position of the recorded bat (stationary); (2) selectivity for the identity of the bat that was taking off; and (3) selectivity for the identity of the bat that was taking off, given the position of the recorded bat (Fig. 3f; see below). The position of the recorded bat was considered as a categorical variable, corresponding to the positional cluster occupied by the recorded bat at the time of take-off of other bats (see above for positional clustering). In all cases, we considered only events with at least ten repetitions of the same class (position or bat identity). Events with less than ten repetitions were discarded, and the selectivity index was calculated considering only the remaining classes. As a result, the selectivity index could be calculated for only those cells in which at least two positions and/or identities remained after exclusion (*n* = 124, 110, 113 respectively). The calculation proceeded as follows: the average response of a cell in a given condition (position or identity) was calculated as the scalar product between the average firing rate in a [−1 s, +1 s] interval around take-off and the template response (see above for magnitude of the response). To calculate a selectivity index, the set of responses across different positions or identities was normalized to assume positive values and the selectivity index was calculated as described previously^71,72^:

$${\rm{SI}}=\left(1-\frac{{\left(\sum \frac{{r}_{i}}{n}\right)}^{2}}{\sum \frac{{r}_{i}^{2}}{n}}\right)\left(\frac{1}{1-\frac{1}{n}}\right)$$

where *n* indicates the number of different conditions (positions or identities) and *r*_*i*_ represents the average response of the cell in that condition. A distribution of 100 shuffled selectivity indexes was calculated by repeating the above steps after random permutation of the positions or the identities. A cell was defined to be significantly selective for a given position or bat identity when the empirical value of the selectivity index exceeded the upper 95% confidence interval of its shuffled distribution. The calculation of the selectivity index for bat identity given position was performed similarly, by systematically considering each position as a separate subset and calculating a selectivity index for bat identity from that subset, therefore obtaining a set of indexes for each cell and position.

### Analysis of calcium imaging data during collective foraging

All behavioural analysis related to the imaging sessions followed the same methods described above, as the structure of the experiment was largely the same in the case of electrophysiology and imaging (6 or 7 bats foraging from a bowl or from four feeders), with the main difference being the shorter duration of imaging sessions (about 60 min). For the analysis of social and spatial responses during imaging, we focused on the subset of sessions for which there was at least one landing location with stereotyped and consistent behaviour of the imaged bat for social versus non-social flights (*n* = 24 FOVs across 20 sessions with one or two simultaneously imaged bats, total of 3 different bats). In particular, we required at least five flights per type (social versus non-social) and no significant differences in the landing position, heading direction (averaged over a [−1, 0] s window before landing) and in the overall movement of the bat (quantified as the absolute deviation of the accelerometer signal from *g*, averaged over a [0, 2] s window after landing) by using a permutation test (*P* < 0.05). Once an analysable landing location was found, we tested each extracted ROI for social modulation by comparing its normalized firing rate (defined above) averaged over a [−1, 2] s time window around landing for social versus non-social flights by using a permutation test (a cell was considered to be socially modulated if *P* < 0.05). For principal component analysis and decoding, we considered as input features the standardized firing rates around social versus non-social landings, averaged over the same [−1, 2] s time window. Each landing was therefore associated with an *N* × *F* matrix, where *N* was the number of socially modulated cells (or a matched number of socially unmodulated neurons) and *F* the number of flights, each one associated with a class (social versus non-social). The first two principal components were used both for visualization (Fig. 4e, left) and for calculating the distance between the activity centroids for social versus non-social flights (Fig. 4e, right). Data from different sessions were pooled and represented in the same principal component plane by rotating all points (social and non-social) from a given session, such that the centroid of activity for social flights would be on the negative PC1 axis. The same method was used for socially modulated as well as unmodulated cells, as a control. For decoding social versus non-social landings, the activity matrix was used to train a logistic regression classifier. Accuracy was calculated using fourfold cross validation. Spatially modulated cells were defined, across the same set of FOVs analysed for social responses, as those ROIs showing significantly different activity when landing on one versus the other of the two most common locations in the room (mean = 31% spatially modulated cells across 24 FOVs, 3 bats), using the same [−1, 2] s time window relative to landing. Spatial decoding was performed as described above by training logistic regression classifiers on the activity matrix, where activity was in this case calculated around landing on the two most common locations in the room. Decoding accuracy—spatial or social—was calculated for different sets of cells (socially modulated, spatially modulated, unmodulated or socially but not spatially modulated). Note that for both social and spatial decoding, the chance level is at 0.5.

### Statistical analysis

No formal methods were applied to predetermine sample sizes and adopted sample sizes were similar to those used by similar studies. No randomization of experimental sessions was performed and no blinding to experimental conditions was implemented during the analysis. All statistical comparisons were performed using nonparametric tests (Wilcoxon rank-sum tests, Wilcoxon signed-rank tests, bootstrap or randomization tests) unless otherwise stated. The tests were two-tailed, unless otherwise stated. Where appropriate, adjustments for multiple comparisons were performed using Bonferroni or Tukey correction.

### Reporting summary

Further information on research design is available in the Nature Portfolio Reporting Summary linked to this article.

## Online content

Any methods, additional references, Nature Portfolio reporting summaries, source data, extended data, supplementary information, acknowledgements, peer review information; details of author contributions and competing interests; and statements of data and code availability are available at 10.1038/s41586-023-06478-7.

### Supplementary information

Reporting Summary

### Source data

Source Data Fig. 1

Source Data Fig. 2

Source Data Fig. 3

Source Data Fig. 4

## Figures and Tables

**Extended Data Table 1:**
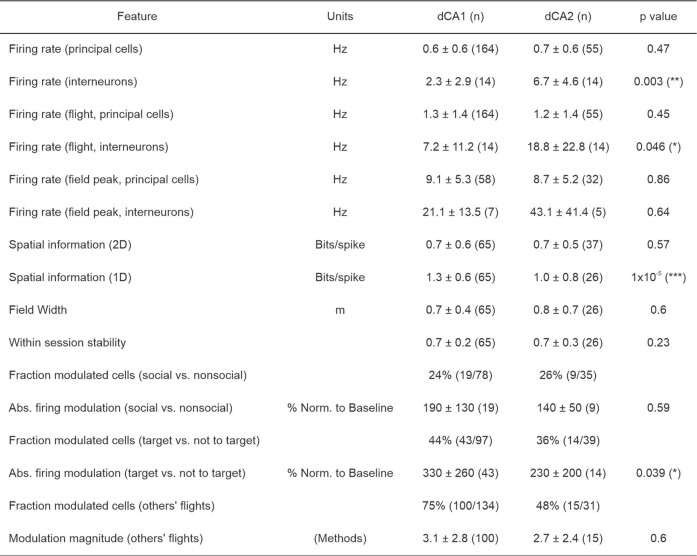
Properties of recorded cells in dCA1 and dCA2

Each row of the table reports different properties of the recorded cells, divided according to the putative location of the recording tetrode in the dorsal hippocampus (Methods). The last column reports the *p-value* of a Wilcoxon rank sum test between the corresponding values in dCA1 and dCA2. Values indicate mean ± standard deviation (or percentages). Numbers in parentheses indicate the number of cells.

## Data Availability

The dataset from this study is available from the corresponding author on reasonable request. Source data are provided with this paper.

## Code availability

All analyses were conducted using custom code in MATLAB (MathWorks). The code generated in the current study is available from the corresponding author on reasonable request.
