# Supplementary material for: Hippocampal representation during collective spatial behaviour in bats
Source: Nature. 2023 Aug 30;621(7980):796–803. doi: 10.1038/s41586-023-06478-7 (PMC10533399; doi:10.1038/s41586-023-06478-7)
Supplement: Supplementary file 1 — Reporting Summary [file 41586_2023_6478_MOESM1_ESM.pdf]

nature portfolio

Corresponding author(s): Michael M. Yartsev

Last updated by author(s): Jul 9, 2023

Reporting Summary

Nature Portfolio wishes to improve the reproducibility of the work that we publish. This form provides structure for consistency and transparency in reporting. For further information on Nature Portfolio policies, see our Editorial Policies and the Editorial Policy Checklist.

Statistics

For all statistical analyses, confirm that the following items are present in the figure legend, table legend, main text, or Methods section.

n/a	Confirmed	
☐	☒	The exact sample size (*n*) for each experimental group/condition, given as a discrete number and unit of measurement
☐	☒	A statement on whether measurements were taken from distinct samples or whether the same sample was measured repeatedly
☐	☒	The statistical test(s) used AND whether they are one- or two-sided<br>*Only common tests should be described solely by name; describe more complex techniques in the Methods section.*
☐	☒	A description of all covariates tested
☐	☒	A description of any assumptions or corrections, such as tests of normality and adjustment for multiple comparisons
☐	☒	A full description of the statistical parameters including central tendency (e.g. means) or other basic estimates (e.g. regression coefficient) AND variation (e.g. standard deviation) or associated estimates of uncertainty (e.g. confidence intervals)
☐	☒	For null hypothesis testing, the test statistic (e.g. *F*, *t*, *r*) with confidence intervals, effect sizes, degrees of freedom and *P* value noted<br>*Give P values as exact values whenever suitable.*
☒	☐	For Bayesian analysis, information on the choice of priors and Markov chain Monte Carlo settings
☒	☐	For hierarchical and complex designs, identification of the appropriate level for tests and full reporting of outcomes
☐	☒	Estimates of effect sizes (e.g. Cohen's *d*, Pearson's *r*), indicating how they were calculated

Our web collection on statistics for biologists contains articles on many of the points above.

Software and code

Policy information about availability of computer code

Data collection	The 3D spatial positions of all the bats were measured through an adaption of a commercially available RTLS system (Ciholas Inc.) and recorded via Python (ver 3.9) scripts (available from the corresponding author on request). Acquisition of neural data was done using a wireless neural logger (Deuteron Technologies, MouseLog16) or an open source, miniature microscope system (https://github.com/gardner_lab/FinchScope). Echolocation calls were recorded via a dedicated ultrasonic microphone (Earthworks Inc.).
Data analysis	Custom MATLAB code was used for all data analysis (MATLAB version 2021a). Imaging video were processed using Imagej (ver 1.53c), registered using MOCO (03-18-206 release, https://github.com/NTCColumbia/moco) and extracted using CNMF-E (https://github.com/zhoupc/CNMF_E). The code generated in the current study is available from the corresponding author on reasonable request.

For manuscripts utilizing custom algorithms or software that are central to the research but not yet described in published literature, software must be made available to editors and reviewers. We strongly encourage code deposition in a community repository (e.g. GitHub). See the Nature Portfolio guidelines for submitting code & software for further information.

Data

Policy information about availability of data

All manuscripts must include a data availability statement. This statement should provide the following information, where applicable:

- Accession codes, unique identifiers, or web links for publicly available datasets
- A description of any restrictions on data availability
- For clinical datasets or third party data, please ensure that the statement adheres to our policy

Data generated and analyzed in this study are available from the corresponding author upon reasonable request.

Human research participants

Policy information about studies involving human research participants and Sex and Gender in Research.

Reporting on sex and gender	NOT APPLICABLE
Population characteristics	NOT APPLICABLE
Recruitment	NOT APPLICABLE
Ethics oversight	NOT APPLICABLE

Note that full information on the approval of the study protocol must also be provided in the manuscript.

Field-specific reporting

Please select the one below that is the best fit for your research. If you are not sure, read the appropriate sections before making your selection.

☒ Life sciences ☐ Behavioural & social sciences ☐ Ecological, evolutionary & environmental sciences

For a reference copy of the document with all sections, see nature.com/documents/nr-reporting-summary-flat.pdf

Life sciences study design

All studies must disclose on these points even when the disclosure is negative.

Sample size	We analyzed a dataset of 373 single-units from 5 bats (electrophysiology), 24 Fields-of-view from 3 bats (imaging) and group behavior from a total of 20 bats. No statistics were used to determine sample size. Sample sizes are similar to those used in the field (e.g. Dotson et al., Science 373.6551 (2021): 242-247. Sarel et al., Nature 609, 119–127 (2022).
Data exclusions	No behavioral sessions were excluded. The inclusion criteria for the neurons were based on sufficient spike number, behavioral coverage and firing stability (Methods), and are standard for this research field. Exclusion criteria were not predetermined.
Replication	Findings on neural activity and structured behavior were replicated across multiple animals, cells and experiments. The reported effects were found across all tested animals (Methods). Experiments consisted of daily sessions during which single-units were collected (typically 1-10 per recorded bat) or neural populations were imaged. No explicit attempt was made to record from the same cells across days.
Randomization	Not relevant, as our study did not involve treatment of experimental groups and no randomization was required.
Blinding	Not relevant, as our study did not involve treatment of experimental groups and no blinding was required.

Reporting for specific materials, systems and methods

We require information from authors about some types of materials, experimental systems and methods used in many studies. Here, indicate whether each material, system or method listed is relevant to your study. If you are not sure if a list item applies to your research, read the appropriate section before selecting a response.

Materials & experimental systems

n/a	Involved in the study
☐	☒ Antibodies
☒	☐ Eukaryotic cell lines
☒	☐ Palaeontology and archaeology
☐	☒ Animals and other organisms
☒	☐ Clinical data
☒	☐ Dual use research of concern

Methods

n/a	Involved in the study
☒	☐ ChIP-seq
☒	☐ Flow cytometry
☒	☐ MRI-based neuroimaging

Antibodies

Antibodies used	Goat anti-Iba1, ab5076, Abcam; Rabbit anti-PCP4, HPA005792, Sigma; Donkey anti-goat Alexa-647, Invitrogen A32849; Donkey anti-rabbit Alexa-488, Invitrogen A-21206.
Validation	Antibodies adopted in this study were validated for routine use in rodents. We followed similar immunohistochemistry protocols for bats.

Animals and other research organisms

Policy information about studies involving animals; ARRIVE guidelines recommended for reporting animal research, and Sex and Gender in Research

Laboratory animals	Fifteen lab-born adult (3-6 years old) male Egyptian fruit bats (Rousettus aegyptiacus) were used in this study.
Wild animals	An additional five wild-caught animals were used as part of the social groups. All of them were male, wild-born and were captured in Israel between 2015 and 2017. Their precise age could not be identified (this species of bats is very long-lived, with a maximum reported longevity of 25 years). Wild-caught animals were socially housed in large colony rooms, together with lab-born animals.
Reporting on sex	All animals involved in this study were males.
Field-collected samples	This study did not involve samples collected from the field
Ethics oversight	All procedures performed in this study were approved by the Institutional Animal Care and Use Committee at the University of California, Berkeley, and were performed in accordance with the Guide for the Care and Use of Laboratory Animals.

Note that full information on the approval of the study protocol must also be provided in the manuscript.
